# *Salmonella* Typhi serine threonine kinase T4519 induces lysosomal membrane permeabilization by manipulating Toll-like receptor 2-Cystatin B-Cathepsin B-NF-κB-reactive oxygen species pathway and promotes survival within human macrophages

**DOI:** 10.1371/journal.ppat.1013041

**Published:** 2025-04-01

**Authors:** Swarnali Chakraborty, Debayan Ganguli, Theeya Nagaraja, Animesh Gope, Sudip Dey, Ananda Pal, Rahul Shubhra Mandal, Sudipta Sekhar Das, Santasabuj Das

**Affiliations:** 1 Department of Clinical Medicine, ICMR - National Institute for Research in Bacterial Infections, Kolkata, West Bengal, India; 2 Division of Infectious Diseases, Washington school of medicine, St. Louis, Missouri, United States of America; 3 Biocon Biologics Limited- R&D centre, Chennai, Tamil Nadu, India; 4 Academy of Scientific and Innovative Research (AcSIR), Ghaziabad, Uttar Pradesh, India.; 5 Department of Cancer Biology, Perelman School of Medicine, University of Pennsylvania, Philadelphia, Pennsylvania, United States of America; 6 Department of Cancer, Apollo Multispeciality Hospitals Limited, Kolkata, West Bengal, India; 7 ICMR-National Institute of Occupational Health, Ahmedabad, Gujarat, India; Shanghai Jiao Tong University Affiliated Chest Hospital, CHINA

## Abstract

Intracellular pathogens of *Salmonella* spp. survive and replicate within the phagosomes, called Salmonella-containing vacuoles (SCVs) inside macrophages by manipulating phagosomal maturation and phagolysosome formation. While controversies exist about the phagosomal traffic of *Salmonella* Typhimurium, little studies were carried out with the intracellular survival mechanisms of *Salmonella* Typhi (*S.* Typhi). We had previously reported that a eukaryote-like serine/threonine kinase of *S.* Typhi (T4519) contributes to survival within macrophages and activates host pro-inflammatory signaling pathways regulated by NF-κB. However, neither the mechanisms underlying NF-κB activation nor how it contributes to intracellular survival of *S.* Typhi were studied. Here we show, by using antibody-mediated blocking and gene knockdown studies that T4519 activates Toll-like receptor 2 (TLR2) signals in the human monocyte-derived macrophages. We computationally predicted the NH2-terminal glycine rich repeat domain of T4519 as the TLR2-binding moiety and confirmed the interaction by co-immunoprecipitation experiment. TLR2-T4519 interaction transcriptionally repressed cystatin B, a cathepsin B inhibitor, leading to the activation of cytosolic cathepsin B, leaked from the lysosomes of the infected cells. Through a series of RT-qPCR, western blotting, gene knockdown, flow cytometry and confocal microscopy experiments, we have shown that active cytosolic cathepsin B cleaves IKB-α, resulting in nuclear translocation of NF-κB and transactivation of its target genes, including reactive oxygen species (ROS), which in turn induces lysosomal membrane permeabilization (LMP). TLR2-dependent targeting of the cystatin B-cathepsin B-NF-κB-ROS pathways by T4519, leading to LMP promotes phagosomal survival of *S.* Typhi. This study describes a unique mechanism of the exploitation of host NF-κB signaling pathways by bacterial pathogens to promote its own persistence within macrophage cells.

## Author summary

Mechanisms behind the persistence of *Salmonella* Typhi, a facultative, intracellular pathogen within human macrophages remain unknown. The type three secretion system 2 effectors, which play critical roles in macrophage survival of *Salmonella* Typhimurium are redundant for *S.* Typhi. We show that T4519, a eukaryote-like serine threonine kinase promotes survival and growth of *S.* Typhi within human primary macrophages. T4519 binds to the Toll-like receptor 2 expressed by macrophages and represses the expression of cystatin B, leading to activation of cathepsin B. The resultant active cytosolic cathpsin B in *S.* Typhi-infected macrophages cleaves inhibitor-κB and translocates NF-κB into the nucleus, where it induces reactive oxygen species (ROS) generation. ROS in turn causes lysosomal damage by membrane permeabilization, promoting *S.* Typhi survival. This is a unique mechanism by which a pathogenic bacterium exploits the protective NF-κB signaling pathways of the host to augment its survival within human macrophages.

## Introduction

Intracellular pathogens, including *Salmonella spp.* survive and grow within the phagosomes of the professional phagocytic cells, especially macrophages. Microbe-containing phagosomes rapidly mature through progressive acidification and acquisition of an elaborate arsenal of microbicidal and degradative molecules, including the reactive oxygen and nitrogen species (ROS and RNS), lactoferrin, NRAMP1, cathepsins, lysozyme and cationic antimicrobial peptides (cathelicidin, defensins) [1,2,3,4,5]. While most of these factors and mechanisms are common to almost all bacterial pathogens, intracellular bacteria are uniquely poised to combat the sophisticated antimicrobial machinery of the phagocytes by arresting or reprograming phagosomal maturation, escaping from the maturing phagosome or resisting antimicrobial properties of phagolysosomes, all of which promote their survival and replication within macrophages [6,7]. Mtb (*Mycobacterium tuberculosis*) arrests phagosomal maturation by employing the membrane lipid lipoarabinomannan (LAM), SapM phosphatase and zinc metalloprotease, ZmpA [8,9,10,11], while *Listeria monocytogenes* uses listeriolysin (LLO) for this purpose [12]. Furthermore, ESX-1-ESAT-6 system of Mtb and LLO--phospholipase C induce phagosomal membrane damage and release the bacteria into the cytosol [13,14,15]. *Legionella pneumophilia* and *Cloxiella burnetii* diverts/ delays lysosomal fusion of the vacuole [16,17]. For *Salmonella* containing vacuoles (SCVs), some studies reported maturation failure caused by the recruitment of syntaxin 8 by type three secretion system 1 (T3SS-1) effector, SipA or by impaired phagsosomal acidification due to the inhibition of aldolase A-mediated v-ATPase assembly [18,19]. Other studies found normal progression of SCV from the early to intermediate phagosome, but phagolysosome formation was inhibited due to delayed LBPA (lysobisphosphatidic acid) accumulation [20] However, *S.* Typhimurium was also reported to replicate within SCVs that had already fused with the lysosomes [21]. One research group observed that that human monocytes and monocyte-derived macrophages readily clears *S.* Typhimurium, but support the survival and growth of *S*. Typhi that does not interfere with phagolysosome formation [22].

Intracellular pathogens may directly target lysosomes of the infected macrophages to avoid killing by the lysosomal enzymes. A study showed that *S*. Typhimurium T3SS2 effector SifA inhibits Rab9-mediated retrograde transport of MPRs (mannose-6-phosphate receptors) to the trans-Golgi network (TGN), preventing traffic of the hydrolases by MPRs to late endosomes and lysosomes. This resulted in the generation of lysosomes with reduced hydrolase content [23]. Further, SUMOylation of SifA prevents lysosomal acidification and promotes bacterial proliferation [24]. Another SPI-2 effector protein, GtgE, a cysteine protease, together with SopD2, binds to and cleaves Rab-32, which augments the biogenesis of lysosomal related organelles (LROs), containing antimicrobial peptides [25]. *Vibrio parahaemolyticus* secrets VapA, a T3SS1 effector protein, which targets V-ATPase subunit c and ruptures host cell lysosomes. [26]. *Streptococcus pneumoniae* toxin pneumolysin causes lysosomal membrane permeabilization (LMP) by activating MyD88 downstream of multiple TLRs, including TLR2 and TLR4, which is independent of the toxin’s pore-forming function [27]. Other TLR signals were also involved in LMP. TLR7 ligand imiquimod induced LMP and caspase-8 mediated apoptosis of skin cancer cells by augmenting ROS production [28]. Multiple other studies have established ROS as a major mediator of LMP [29]. Intriguingly, *TLR2*^−/−^ macrophages infected with *S*. Typhimurium had lower bacterial load compared with the wild type cells, due to the upregulation of inducible nitric oxide synthase (iNOS) expression and reduced proinflammatory cytokine levels [30].

Eukaryote-like serine/threonine kinases (eSTKs) of the prokaryotes sense environmental cues and regulate multiple physiological functions [31]. For intracellular pathogens, eSTKs were also shown to promote virulence by targeting host molecules and signalling pathways. *M. tuberculosis* PknG inhibits phagosome maturation by sequestering host cell PKCα [32] or by blocking Rab7 recruitment to the phagosomal membrane through the inhibition of Rab7L1-GTP formation [33]. In contrast, *C. burnetii* CstK regulates the maturation process and bacterial survival by interacting with TBC1D5, a Rab7 GTPase-activating protein [34]. Several eSTKs target the host innate immune signalling pathways and modulate pro-inflammatory cytokine production. LegK1 of *L. pneumophilia* activates NF-κB signalling cascade by phosphorylating the inhibitors, IκB and p100 and induces pro-inflammatory cytokines [35]. On the other hand, *Shigella* OspG and Mtb PknG inhibit NF-κB by two different mechanisms, namely sequestration of ubiquitinylated E2 enzymes, UbcH7 and UbcH5, leading to the failure of proteasome-mediated IκBα degradation [36] and ubiquitination and degradation of TRAF2 and TAK1 through E3 ubiquitin ligase function [37], respectively. Both LegK2 of *Legionella pneumophilia* and *S.* Typhimurium SteC influence global cytoskeleton remodelling; LegK2 through phosphorylation of ARPC1B/APR3 subunits of the ARP2/3 complex [38] and SteC by activation of the MAPKs (MEK and ERK)-myosin light chain kinase-myosin IIB signalling pathway [39]. However, given the functional redundancy of the eSTKs, which are usually present in multiples in a single organism, it is often difficult to identify the specific role of an individual eSTK in the pathogenesis.

While the mechanisms underlying intracellular survival of non-typhoidal *Salmonella*, such as *Salmonella* Typhimurium (*S*. Typhimurium) have been extensively studied, similar reports for *S*. Typhi are scanty. Despite the two *Salmonella* serovars being closely related, having 98%-99% sequence identity at the nucleotide level, their virulence gene sequences, encoded by the pathogenicity islands display nearly 11% difference [40]. Importantly, survival of *S*. Typhimurium within phagocytes requires T3SS-2 and its secreted effector proteins, encoded by a genomic island, called *Salmonella* Pathogenicity Island 2 (SPI-2) [41]. In contrast, *S*. Typhi does not require T3SS-2 or the SPI-2 genes, many of which have turned into pseudogenes in *S*. Typhi for intracellular survival [42,43]. The above factors perhaps explain different human diseases, namely enterocolitis and systemic febrile illness caused by *S*. Typhimurium and *S*. Typhi, respectively.

We had earlier reported the first eSTK of *S*. Typhi, called T4519 that is needed for bacterial survival inside macrophages and pathogenesis in a mouse model [44]. NCBI BLAST search revealed T4519 amino acid sequences homologous to those of the eukaryotic protein kinases over the kinase catalytic domains. Similarity searches of T4519 sequence with the known crystal structures from the Protein Data Bank (PDB) showed *M. tuberculosis* PknB topping the list. Most importantly, 11 of the 12 critical amino acid residues that define the eukaryotic protein kinase superfamily are conserved in T4519 [44]. The activation loop is present between the conserved DFG (Asp-Phe-Gly) and APE (Ala-Pro-Glu) motifs. The conserved RD (arginine-aspartate) sequence in the catalytic loop of eukaryotic kinases, which interacts with the phosphorylated Ser/Thr residues in the activation loop and stabilizes the activation loop in a conformation that allows substrate binding and catalysis, is also conserved in T4519. In addition, one STK active-site signature {[LIVMFYC]-x-[HY]-x-D-[LIVMFY]-K-(2)-N-[LIVMFYCT](3)}, as mentioned in the PROSITE database (Prosite entry PS00108) was identified in the T4519 sequence. The gene encoding the putative eSTK of *S*. Typhi (*prkX* [*t4519*] is aligned in tandem with another eSTK (*prkY* [*t4520*]) and a Ser/Thr phosphatase 2C gene (*prpZ* [*t4521*]), forming the *prpZ* gene cluster, which is exclusively present in *S*. Typhi and absent from all other *Salmonella* serovars. T4519 was found to be induced by ROS within the phagosome, followed by translocation into the cell cytosol and modulate innate immune responses. In the present study, we have explored the underlying mechanisms of how T4519 contributes to *S.* Typhi survival within macrophages. We found that, T4519 activates cytosolic cathepsin B (Cat B) through TLR2-dependent downregulation of the default inhibitor protease, cystatin B (stephin B). Activated cathepsin B cleaves IκB-α, leading to nuclear import of NF-κB. We further showed that Cat B-NF-κB pathway induces ROS and ROS-dependent lysosomal membrane permeabilization (LMP), promoting bacterial survival.

## Results

### T4519 promotes delayed and proteasome-independent IκB-α degradation and p65 nuclear translocation in human monocyte-derived macrophage (MoM) cells

We had earlier reported that a eukaryote-like serine/threonine kinase (T4519) of *S*. Typhi promoted bacterial survival in macrophages, derived from human myelomonocytic cell line THP-1 (THP-1 derived macrophages or TDM). We further demonstrated that T4519 is a secreted kinase that induces degradation of IκB-α and nuclear translocation of NF-κB p65 [44]. Here, we investigated the detail mechanisms underlying IκBα degradation and NF-κB nuclear translocation by T4519 and its role, if any in *S*. Typhi survival within human primary macrophage cells. To this end, we generated macrophages from the peripheral blood monocytes (monocyte-derived macrophages or MoM) by the treatment of M-CSF (Macrophage Colony-Stimulating Factor) and infected them with wild type *S*. Typhi Ty2 strain (Ty2) or its isogenic deletion mutant lacking T4519 (Ty2∆t4519). Similar to the TDM infection, both the wild type and mutant bacteria were equally efficient to infect MoM cells. However, intracellular bacterial counts were reduced after the cells were cultured for 24h, as opposed to increased numbers in TDM, suggesting that human primary macrophage cells were more efficient in limiting the intracellular growth of *S*. Typhi. But, the reduction was only modest for the Ty2 strain as compared with markedly reduced count of Ty2∆t4519 (S1 Fig). This result indicated that T4519 contributed to intracellular survival of *S*. Typhi Ty2 in human primary macrophages. An LDH assay to assess macrophage viability ruled out that the above results could be due to differential cell death induced by the two strains (S2 Fig). We further standardized that the numbers of live intracellular bacteria recovered 24hrs post-infection were equalized for the wild type and mutant *S*. Typhi strains when the MoI for the latter was made double that of the wild type bacteria (S3 Fig). To prove that our results were determined by the presence or absence of T4519 rather than the number of bacteria, we infected the MoM cells with twice as many of Ty2∆t4519 as the Ty2 strain for our subsequent experiments except for the bacterial intracellular survival studies where the same MoI was used for the wild type and the mutant strains to evaluate the survival advantage conferred by T4519.

**Fig 1:**
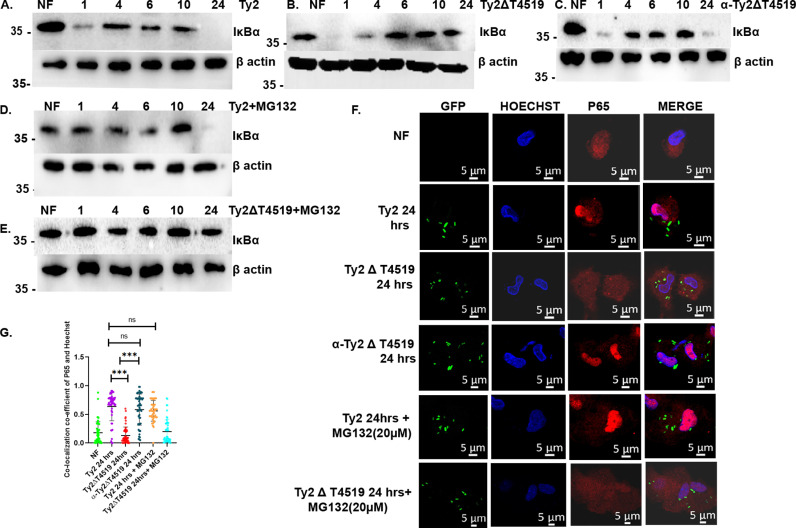
T4519 promotes delayed IκBɑ **degradation followed by p65 nuclear translocation independent of proteasomal degradation of I κ B α in human MoM cells.** MoM Cells pre-treated with proteasome inhibitor (MG132, 20 µM) 3 hrs prior to infection with wild type *S*. Typhi Ty2, Ty2ΔT4519 or complemented mutant (α- Ty2ΔT4519) strain for 30 mins, followed by gentamycin protection assay as described under Materials and Methods. NF stands for no Infection. A-E. Western blots for IκB-α. The numbers denoted hrs after infection. The experiments were repeated three times and one representative blot in each case is shown here. β-actin was used as the loading control. (F). Confocal micrographs of MoM cells infected with GFP-tagged bacteria and stained with p65 primary antibody, followed by Alexa-fluor 594 conjugated anti-rabbit secondary antibodies (red). Hoechst dye (blue) was used to stain the cell nucleus. Images were taken in 63X oil immersion lens of LSM 710 confocal microscope; scale bar 5µm. Representative images from three independent experiments are shown. Error bars represent SD. Statistical significance was calculated by two tailed unpaired t test using GraphPad Prism version 8. (G). Fluorescence colocalization of Hoechst (blue) and p65 (red) was quantified in 50 cells from randomly taken fields using Zen Blue software of LSM 710 confocal microscope (Zeiss). ***, p< 0.001, compared between Ty2 and Ty2ΔT4519 or α-Ty2ΔT4519 and Ty2ΔT4519 infected cells at 24hrs time point.

- To investigate if the canonical pathway was involved in NF-κB activation by T4519, we first examined IκB-α degradation in the infected cell lysates by western blots. The results showed biphasic IκB-α degradation in Ty2-infected cells, while Ty2∆t4519 only induced the early IκB-α degradation (Figs 1A, 1B and S4A). That T4519 could function standalone to promote IκB-α degradation at 24hrs was proved by similar degradation pattern for Ty2 and the complemented strain (α-Ty2ΔT4519) (Fig 1C). Next, the involvement of proteasome in IκB-α degradation was studied by pre-treatment of the cells with proteasome inhibitor (MG132), followed by infection with Ty2 or Ty2∆t4519. Intriguingly, we found that T4519-independent early degradation of IκB-α, but not the late degradation induced by T4519 was proteasome-mediated (Figs 1D, 1E and S4A). That the late IκB-α degradation by T4519 also led to NF-κB nuclear translocation was proved by staining of the *S*. Typhi-infected MoM cells with p65 antibodies. p65 was predominantly present in the nucleus after 24 hrs of Ty2 and α-Ty2ΔT4519 infection as opposed to largely cytosolic localization in the un-infected and Ty2∆t4519-infected cells (Fig 1F). This resulted in significantly higher co-localization co-efficient of the red and blue fluorescence, used for the staining of p65 and the cell nucleus, respectively in Ty2- and α-Ty2ΔT4519 infected cells compared with the uninfected and mutant bacteria-infected cells (Figs 1F and S4B). However, p65 nuclear translocation at the early time point was observed after both Ty2 and Ty2∆t4519 infection. Corroborating the above findings, p65 nuclear translocation after 1hr (S4D–S4F Fig), but not after 24hrs of infection was proteasome-dependent (Figs 1F, 1G and S4D–S4F). On the other hand, *S*. Typhimurium LT2 strain failed to promote p65 nuclear translocation in the infected cells at 24hrs P.I. (S4C Fig).

### Delayed, but not early IκB-α degradation and p65 nuclear translocation in *S*. Typhi-infected human MoM cells are cathepsin B dependent

**Fig 2:**
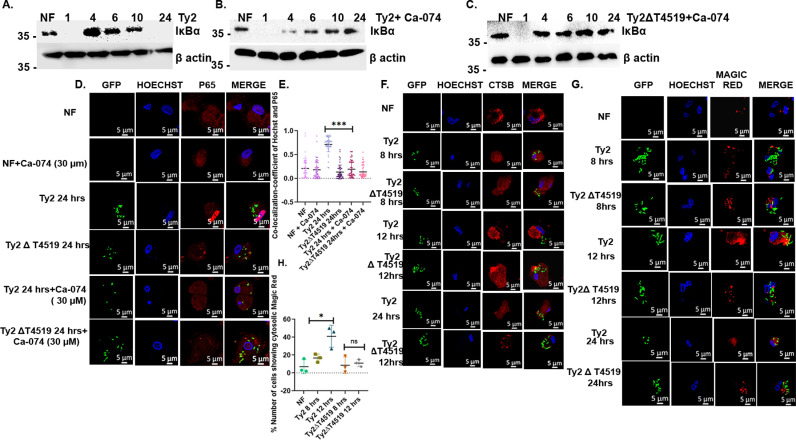
Delayed , **but not early IκB-α** **degradation and p65 nuclear translocation in *S.*** **Typhi-infected human MoM cells are cathepsin B dependent.** MoM cells were treated with cathepsin B inhibitor (Ca-074, 30 µM) 3 hrs prior to infection. (A-C). Western blots of the infected cell lysates probed with IκBα. β-actin was used as a loading control. The experiments were repeated three times and one representative blot from each experiment is shown here. (D-E). Confocal microscopy images of cells pre-treated with or without cathepsin B inhibitor and stained for p65 as under Fig 1F above. (D). Representative images from three independent experiments are shown. (E). Quantification of fluorescence colocalization of Hoechst (blue) and p65 (red) as performed under Fig 1G above. ***, p< 0.001, as compared between cathepsin B inhibitor untreated and treated cells after 24hrs of infection with Ty2 strain. (F). Cathepsin B antibody staining of MoM cells infected for various time points. Alexa-fluor 594 conjugated anti-rabbit secondary antibodies were used. (G). MoM cells infected as indicated with GFP-expressing bacteria and stained with Cathepsin B substrate, Magic Red. Excitation done at 594nm (red). Nucleus was stained with Hoechst (blue). Representative confocal microscopic images from three independent experiments are given. (H). Quantification of magic red fluorescence in the cell cytosol of randomly selected 50 cells from different fields was done. *, p<0.05. Error bars represent SD. Significance was calculated using two tailed unpaired t test. Statistical analysis was done using GraphPad Prism version 8.

Published studies suggested that cathepsin B can induce proteasome-independent IκB-α degradation, leading to NF-κB activation [45,46]. Given that T4519-regulated IκB-α degradation was proteasome-independent, we investigated if this was mediated by cathepsin B. To this end, MoM cells were treated with highly selective cathepsin B inhibitor, CA-074, which is an epoxysuccinyl peptide derivative of E-64 with cathepsin B inhibition at nanomolar concentrations, followed by *S*. Typhi infection. Probing the cell lysates for IκB-α in western blots showed complete blockade of degradation after 24hrs of Ty2 infection in the inhibitor-treated cells, but early IκB-α degradation was spared (Figs 2A–2C, and S5A). Accordingly, nuclear translocation of p65 at 24 hrs, but not at 1 hr post-infection (p.i.) was completely abrogated by cathepsin B inhibitor pre-treatment of the Ty2-infected cells (Figs 2D, 2E, and S5D). Together the above results suggested that T4519 stimulated cathepsin B to promote IκB-α cleavage, followed by p65 nuclear translocation in human MoM cells only at the later time point of infection. This would require active cathepsin B in the cytosol that is normally absent, but may be released from the damaged lysosomes. However, minor damages are successfully repaired, while the enzymatic activity of the released cathepsin B is suppressed by the default cytosolic cathepsin B inhibitor, cystatins. To investigate if active cathepsin B was present in the cytosol of Ty2-infected macrophages, we first stained the infected MoM cells with cathepsin B antibody at different time points. The results showed diffuse Cathepsin B staining of the cytosol after both the wild type and mutant bacterial infections during 8–12 hrs post-infection period (Fig 2F). This was corroborated by cathepsin B western blots of the cytosolic extracts from the above cells (S6 Fig). These results indicated early lysosomal damage of the infected cells. Further to investigate if the cytosolic cathepsin B was enzymatically active, cells were stained with Magic Red, a cathepsin B substrate. We observed speckled Magic Red staining pattern for both wild type and mutant bacterial infections at the early hrs (8 hrs p.i.), representing exclusively lysosomal active cathepsin B (Fig 2G and 2H). Together these results suggested the presence of inactive cathepsin B in the cytosol of the infected cells. However, at 12hrs post-infection (p.i.), strong cytosolic Magic Red staining, suggestive of enzymatic activity was observed in the Ty2-, but not in the Ty2∆t4519-infrected cells (Figs 2G, 2H, and S7).

### T4519-induced cathepsin B activation, IκB-α degradation and p65 nuclear translocation are cystatin B dependent

**Fig 3:**
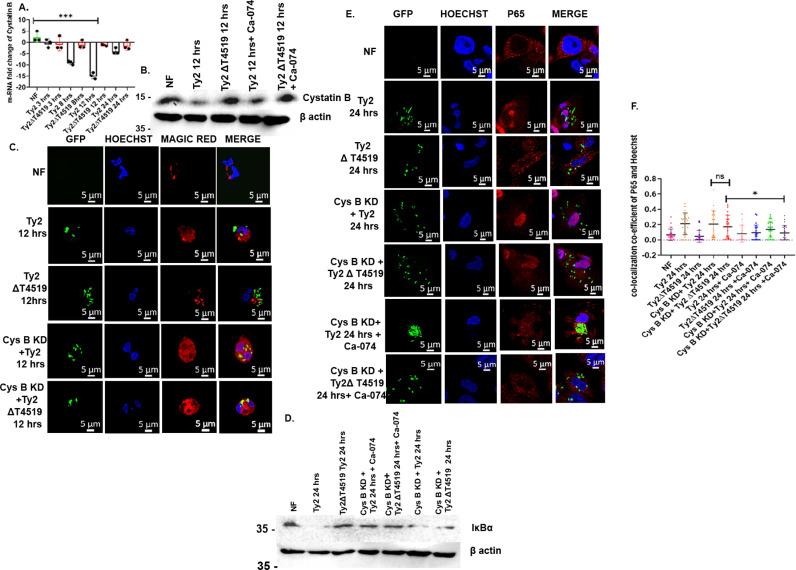
Cathepsin B activation and cathepsin B dependent delayed IKB **α** **degradation and p65 nuclear translocation are mediated by cystatin B.** (A). m-RNA expression of cystatin B measured by RT-qPCR in MoM cells, ***, p<0.001 as compared between non-infected and 12hrs Ty2-infected cells. (B). Western blots of cystatin B in MoM cell lysates. β-actin was used as a loading control. (C). Confocal microscopy images of TDM cells with or without silencing of cystatin B gene expression using siRNA and infected as indicated. Cells were stained for p65 as under Fig 1F above and viewed under the microscope as described under Fig 2G. r . Representative images from three independent experiments are shown. Scrambled siRNA was used as a transfection control. (D). Western blots of IκBα from the parental and cystatin B knock down TDM cells. β-actin was used as a loading control. One representative blot from three independent experiments is shown. (E). Parental and cystatin B knock down TDM cells, infected as indicated were stained and viewed as described under Fig 1F above. (F). Fluorescence quantification of the cells was done as described under Fig 1G by randomly selecting 25 cells from different fields. *, p<0.05 compared between cystatin B gene-silenced TDM cells, pre-treated with or without cathepsin B inhibitor and infected with Ty2Δt4519 for 24 hrs.

To address the underlying mechanisms behind T4519-dependent cytosolic cathepsin B activation, we checked for mRNA expression levels of cystatin B and C, which are default cellular inhibitors of cytosolic cathepsin B [47]. We observed progressive reduction of cystatin B mRNA expression levels in the Ty2-infected cells, reaching nearly 16-fold lower compared with the uninfected cells after 12hrs of infection. In contrast, minimal changes in the cystatin B levels were noticed following infection with the mutant *S.* Typhi (Fig 3A) as well as in *S.* Typhimurium (S8 Fig). Cystatin B protein expression in the cells matched with the mRNA expression levels and showed reduction after Ty2, but not after Ty2∆t4519 infection (Fig 3B). Pre-treatment of the cells with cathepsin B inhibitor had no effects on cystatin B expression, suggesting that it was not under the control of cathepsin B (Fig 3B). In contrast, cystatin C expression remained unaltered after infection with Ty2 or Ty2∆t4519 (S9A and S9B Fig). Given the reciprocal relations between cystatin B and active cytosolic cathepsin B levels in the cells, we investigated if Ty2 infection regulated cathepsin B activation in the cytosol after altering cystatin B levels. To this end, we wanted to check cathepsin B activity after silencing cystatin B with siRNA (S10 Fig). Staining of the cells with magic red showed marked cytosolic cathepsin B activity in both Ty2 and Ty2∆t4519 strain-infected cells (Fig 3C). This suggested that T4519 inhibited cystatin B expression, leading to the activation of cytosolic cathepsin B.

Next, we investigated if T4519-dependent suppression of cystatin B expression could result in IκBα degradation and NF-κB nuclear translocation through cathepsin B activation. To this end, cystatin B gene-silenced TDM cells were pre-treated with or without cathepsin B inhibitor, followed by *S*. Typhi infection. In agreement with the above results, IκBα was degraded in the parental, Ty2- as well as mutant bacteria-infected, cystatin B gene-silenced cells. However, inhibition of cathepsin B in these cells completely restored IκBα levels, suggesting that cystatin B downregulation activated cathepsin B that was responsible for IκBα degradation (Fig 3D). That cystatin B downregulation also activated NF-κB in a cathepsin B-dependent way was proved by confocal microscopy that showed nuclear translocation of NF-κB in the Ty2-infected as well as cystatin B gene-silenced cells, which had active cytosolic cathepsin B, but not in the cathepsin B inhibitor pre-treated cells (Fig 3E). Consequently, co-localization co-efficient of p65 and the nucleus was significantly higher in the cells having active cathepsin B in the cytosol (Fig 3F). These results suggested that T4519 downregulated cystatin B expression levels to activate cathepsin B-NF-κB axis in the MoM cells. However, this phenomenon was a rather late event (beyond 12 hrs of infection), while cystatin B expression remained unaltered in the early hrs of infection (Fig 3A).

### NF-κB activation by cystatin B-cathepsin B axis regulated pro-inflammatory cytokines and ROS

**Fig 4:**
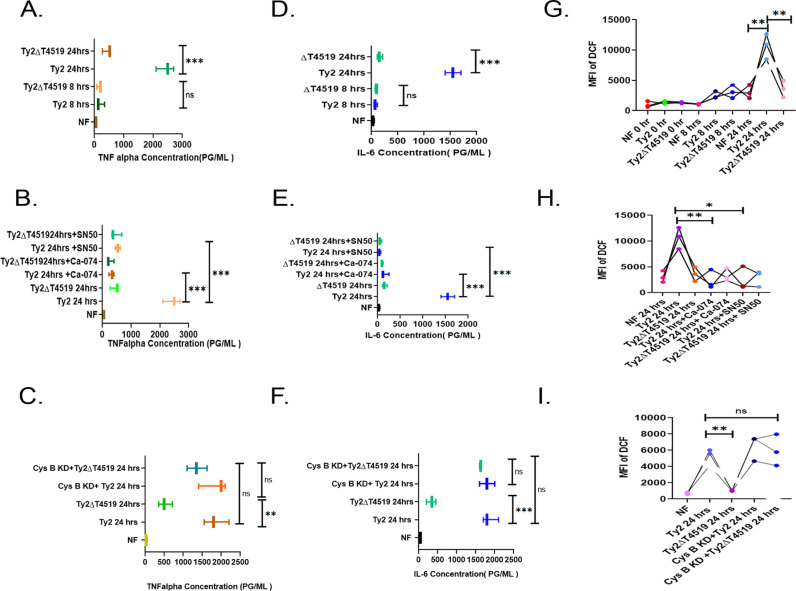
Cystatin B- and cathepsin B-dependent NF- **κB activation regulates pro-inflammatory cytokines expression and ROS generation.** (A, C). TNF-α and IL-6 measured by ELISA in the culture supernatants of MoM cells infected with Ty2 or Ty2ΔT4519 as indicated. (B, D). MoM cells were pre-treated with different inhibitors followed by infection with Ty2 or Ty2ΔT4519 for 24hrs and TNF-α and IL-6 were measured as described above. (C, F). The above cytokines were measured as described above in the culture supernatants of TDM cells. All the above experiments were repeated three times and the values from all three experiments were plotted. Error bars represent SD. Significance was calculated using two tailed unpaired t test. Statistical analysis was done by using GraphPad Prism version 8. NF stands for not significant. ***, p< 0.001; **, p< 0.01. (G-I). ROS generation was measured by flow cytometer (FACS Arya II, BD at FITC channel) in MoM (G, H) or TDM (I) cells after pre-treatment with different inhibitors and infection with Ty2 or Ty2Δt4519 as indicated. (G). **, p< 0.01, Ty2 infection compared with no infection and Ty2 infection compared with Ty2ΔT4519 for 24hrs. (H). **, p<0.01 compared between cathepsin B uninhibited and inhibited ,Ty2 infected cells for 24hrs; *. p<0.05, compared between cells infected with Ty2 for 24hrs after NF-kB nuclear translocation was inhibited or not inhibited.

Previous studies indicated the release of pro or anti-inflammatory cytokines from the infected macrophage cells by intracellular bacterial pathogens. A recently published article reported replicating *S*. Typhi to preferentially choose pro-inflammatory M1 macrophages [48]. We investigated if Ty2 and Ty2∆t4519 differentially stimulated pro-inflammatory cytokines and ROS production in MoM cells. In contrast to rapid induction of cytokines by *S.* Typhimurium [49], *S.* Typhi strain minimally induced TNF-α and IL-6 after 8hrs of infection. However, Ty2, but not Ty2∆t4519 significantly augmented pro-inflammatory response such as TNF-α, IL-6 and ROS production after 24hrs (Figs 4A, 4D, 4G, and S11A). This suggested that T4519 contributed to late cytokine and ROS induction in *S*. Typhi-infected human macrophages. On the other hand, T4519 did not contribute to the production of IL-10, an anti-inflammatory cytokine (S11B Fig). Next, we investigated if the pro-inflammatory mediator release might be regulated by cystatin B-cathepsin B-IkBα-NF-κB pathways, activated by T4519. We observed failure of cytokine surge and ROS production when cathepsin B activity was inhibited in Ty2-infected cells (Figs 4B, 4E, 4H, and S11B) or p65 nuclear translocation was abrogated by SN-50 (Figs 4B, 4E, 4H, S11D and S11B). In contrast, TNF-α, IL-6 and ROS were also induced in the mutant bacteria infected cells when cystatin B expression was silenced (Figs 4C, 4F, 4I, and S11C), indicating a crucial role for cystatin B downregulation-dependent cathepsin B activation, leading to IκBα cleavage and NF-κB nuclear translocation in the regulation of pro-inflammatory cytokines and ROS production after 24 hrs of Ty2 infection.

### Cystatin B-Cathepsin B-NF-κB axis induces LMP through ROS generation

**Fig 5:**
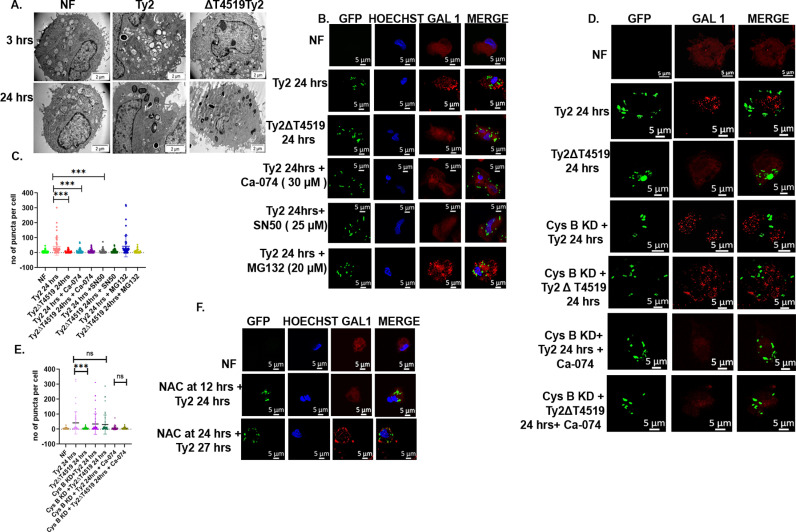
Cystatin B-Cathepsin B-NF- **κB induces LMP through ROS generation.** MoM cells were pre-treated with different inhibitors (B-C) or cystatin B gene expression was silenced in TDM cells (D-E), followed by infection with Ty2 or Ty2∆t4519 for 24hrs. (A). Representative images from transmission electron microscopy. Black unsolid and white solid arrowheads indicate lysosomes (containing BSA-gold particles) and bacteria within phagolysosomes, respectively. Micron bars are shown at the lower right corners. (B). Twenty-four hours after the infection, MoM cells were stained with Galectin 1 antibody (Red), followed by Alexa-fluor conjugated secondary antibodies, while the nucleus was stained with Hoechst (blue). The images were captured as described under Fig 1F. Representative images from three independent experiments are given. (C). Quantification of Galectin-1 puncta per cell, enumerated by Fiji ImageJ software particle analysis tool. Signals from non-puncta were eliminated manually. Cells were randomly selected from each field. N=50 cells. ***, p< 0.001 compared between Ty2 and Ty2ΔT4519 infection or Ty2 infection with and without cathepsin B or NF-kB inhibitor pre-treatment. The images were captured and processed as described under Fig 1G. (D-E). Parental and cystatin B gene silenced TDM cells were infected with Ty2 or Ty2ΔT4519 strain for 24hrs. Cells were stained, images were captured and fluorescent Galectin 1 puncta were counted as describe under (B-C) above. Representative images from one out of three independent experiments are shown. ***, p< 0. 001, compared between Ty2 and Ty2ΔT4519 post-infection at 24hrs. (F). MoM cells, infected and treated with ROS inhibitor (NAC, 1mM x 3hrs), followed by galectin-1 antibody staining as described above. Error bars in the graph represent SD and significance was calculated using two tailed unpaired t test. Statistical analysis was done using GraphPad Prism version 8.

Intracellular pathogen *Salmonella* survives and replicates inside a modified phagosome, known as *Salmonella*-containing vacuole (SCV) within macrophages. We investigated if the signalling pathways activated by T4519, as described above contributed to phagosomal survival of *S*. Typhi. Manipulation of phagosomal acidification and phagolysosome formation is a common survival mechanism of different intracellular pathogens. We measured pH of the acidic organelles of MoM cells after infection with the wild type and mutant *S*. Typhi strains by staining the cells with lysosensor yellow-blue DND [50]. The pH started rising for both infections as early as 3h, but the vacuolar pH of the Ty2 infected cells eventually rose beyond 7.0 after 24hrs of infection, whereas it stopped at pH 5.5 after Ty2Δt4519 infection (S12A and S12B Fig). Next, we investigated by transmission electron microscopy if T4519 exerts any influence on phagosome-lysosome (P-L) fusion. Phagolysosome formation was seen as early as 3hrs of infection, but the numbers of lysosome-like structures were significantly reduced in Ty2-infecetd cells 24hrs post-infection. In contrast, the lysosome like structures were largely retained in the Ty2Δt4519 infected cells (Fig 5A). To investigate if this happened in the Ty2 infection due to LMP, we stained the cells with galectin-1 antibody, which yields diffuse cytosolic staining in presence of intact lysosomal membrane, but a speckled pattern due to the entry of galectin 1 into the lysosome when membrane integrity is compromised. While the un-infected and 1 hr infected cells showed diffuse galectin-1 staining (S13A–S13C Fig), prominent galectin 1 puncta, indicative of LMP was observed after 24hrs of Ty2, but not Ty2Δt4519 infection (Figs 5B and S13D). Further, pre-treatment of the 24hrs Ty2-infected cells with cathepsin B and p65 nuclear translocation inhibitors abrogated the speckled staining pattern, while proteasome inhibitor pre-treatment exerted no effects (Figs 5B, 5C, and S13E). As expected, none of the inhibitors produced any change in the diffuse staining pattern of Ty2Δt4519-infected cells (S13F Fig). Dextran Alexa-fluor staining of the acidic vacuoles in the parallel experiments corroborated with the above findings, showing lysosomal puncta in the cells, except those infected with Ty2 for 24h, which showed diffuse cytosolic staining that was abrogated by pre-treatment with cathepsin B or p65 nuclear translocation inhibitors (S14A–S14E Fig). Lysotracker red staining showed decreased numbers of lysosomal puncta in Ty2 infected parental, but not p65 knockdown TDM cells at 24 hrs p.i. (S14F Fig). Together these results indicated that Ty2 infection of MoM cells for 24hrs led to T4519-induced LMP, mediated by cathepsin B-dependent, but proteasome-independent NF-κB activation.

Our earlier experiments showed that T4519 activated cytosolic cathepsin B by down-regulating cystatin B expression, thereby inducing IκBα cleavage and nuclear translocation of NF-κB. We investigated if T4519-elicited LMP, regulated by cathepsin B-NF-κB axis also depended on cystatin B expression. To address this issue, we infected the TDM cells with the wild type or mutant Ty2 strain for 24hrs after silencing cystatin B expression. Staining with galectin-1 antibody showed prominent puncta in the cystatin B knockdown cells after Ty2Δt4519 infection that was identical to the Ty2-infected cells, suggesting LMP induction. However, inhibition of cathepsin B abrogated the effects of cystatin B gene silencing (Figs 5D, 5E, S15A and S15B). Similar experiments, followed by dextran Alexa-fluor staining of the cells corroborated with the above findings, with cystatin B knockdown resulting in diffuse cytosolic staining pattern after Ty2Δt4519 infection that was similar to Ty2 infection and suggested LMP (S15C–S15D Fig). Together these results underscored that cystatin B down-regulation by T4519 was indeed responsible for the induction of LMP after Ty2 infection, which was mediated by cathepsin B-NF-κB axis (Fig 5D). As ROS is known to cause LMP [29] and was induced by T4519 in MoM cells, we investigated if ROS production underlied LMP induction. Scavenging ROS 12 hrs post infection completely inhibited LMP in Ty2-infected cells, but the effects were not observed when ROS was scavenged after 24 hrs (Fig 5F). Taken together, these findings revealed ROS-mediated LMP by T4519 downstream cystatin B suppression, leading to the activation of cathepsin B- NF-κB axis.

### T4519 functions as TLR2 ligand

**Fig 6:**
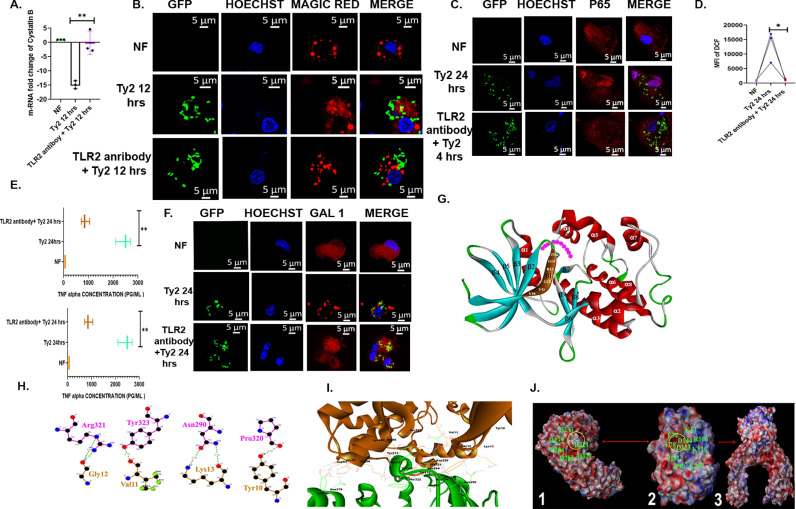
T4519 acts as a TLR2 ligand. MoM cells were incubated with TLR2 antibody for 5hrs followed by infection with Ty2 for different durations as indicated. (A). Cystatin B m-RNA expression as measured by RT-qPCR after blocking TLR2 with antibodies, **, p< 0.01. (B). Antibody pre-treated and infected cells as above were stained with Magic red and viewed under confocal microscope as described under Fig 2G. (C). Confocal microscopy images of cells stained with p65 antibodies after pre-treatment with TLR2 antibodies and infection with Ty2. (D). ROS generation in the above cells were measured by flow cytometer (FACS Arya II, BD) in the FITC channel after blocking of TLR2 receptor, followed by Ty2 infection. *, p<0.05. (E). TNF-α and IL-6 concentrations in the culture supernatants of MoM cells were measured by ELISA. (F). Galectin-1 antibody stained ,infected MoM cells were viewed under confocal microscope as described under Fig 5B. Error bars represent SD. Significance was calculated using two tailed unpaired t test. Statistical analysis was done by using GraphPad Prism version 8. (G). Homology model of T4519 showing substrate binding pocket (pink motifs) and the residues from glycine-rich loop (marked in brown and labeled in white) involved in H-bond interaction with TLR2. (H). H-bond interaction between glycine-rich loop of T4519 (brown) and TLR2 (pink). Hydrogen bonds are indicated by dashed green lines, with bond lengths shown in Å. (I). Overall H-bond (blue) interactions between T4519 (brown) and TLR2 (green). (J). Electrostatic surface representation of TLR2(1), T4519(2) and the complex (3). Yellow circles represent positively-charged hinge region of TLR2 (surface marked in blue) and negatively charged groove of T4519 (surface marked in red) and the corresponding amino acids are labeled. Yellow dotted lines represent the distribution of the corresponding charged residues.

Our results thus far have shown that T4519 promotes bacterial survival and replication within macrophages and activates NF-κB. Since TLRs constitute the major family of microbial recognition receptors in macrophages, we investigated if T4519 functions as a TLR ligand to promote *S*. Typhi survival in MoM cells. To this end, TLRs expression in TDM cells were silenced with si-RNAs, followed by infection with Ty2 for 24 hrs and intracellular CFUs were counted. The results showed significantly reduced recovery of live bacteria from the cells silenced for TLR2, but not after other TLRs expression, suggesting that TLR2 played a critical role for Ty2 survival. (S16A Fig). This was supported by nearly equal recovery of the wild type and mutant strains when TLR2 was blocked by antibodies, suggesting that TLR2-mediated survival of *S.* Typhi in MoM cells was dependent on T4519 expression (S16B Fig). To address if TLR2 transduced signals through Cystatin B-cathepsin B-NF-κB-ROS-LMP pathway, cystatin B m-RNA expression was checked in the untreated and TLR2 antibody pre-treated MoM cells, infected with Ty2. Cystatin B downregulation was abolished in presence of TLR-2 antibody, suggesting that cystatin B down-regulation by T4519 was TLR2-mediated (Fig 6A). To probe the underlying mechanism of cystatin B down-regulation, we checked for the regulation of gene silencer elements by TLR2 signals. Literature searched revealed that out of several cystatin B gene silencers, TLR2 promoted the expression of three splicing factors, namely NOVA-1, SRSF3 and YB-1 through its interaction with the Wnt signalling pathway [51]. We studied the temporal profile of NOVA-1 and YB-1 m-RNA expression in Ty2-infected cells that showed 3–7-fold increase of YB-1 m-RNA at 12 hrs P.I., but no changes in NOVA-1 expression. (S17A and S17B Fig). However, YB-1 expression dropped to the level similar to the non-infected cells if the cells were treated with TLR2 antibody before the infection (S17B Fig). These results suggested that T4519-induced cystatin B down-regulation could be mediated by the induction of YB-1 downstream of TLR2 activation. Further, blocking of TLR2 by antibodies resulted in the absence of active cytosolic cathepsin B (Fig 6B) as well as the abrogation of p65 nuclear translocation, ROS production and pro-inflammatory cytokine induction (Fig 6C, 6D and 6E) in the Ty2-infected cells. We studied by confocal microscopy if lysosomal damage induced by T4519 was regulated by TLR2 signals. We observed significantly reduced staining of the lysosomes when T4519 was expressed in the parental, but not in TLR2 knockdown cells, suggesting involvement of TLR2 signals in lysosomal damage (S18 Fig). This was further corroborated by the absence of Galectin-1 puncta in the cells blocked for TLR-2 signals, suggesting absence of LMP after Ty2 infection (Fig 6F).

To study the interactions between T4519 and TLR2 in further detail, 3D homology model of T4519 was constructed using the crystal structure of PknB (PDB ID: 1O6Y), a ser/thr kinase of *Mycobacterium tuberculosis*, bearing significant sequence homology to T4519 as the template [44]. The model structure conformed to the conserved internal architecture of a protein kinase [52]. 3D model structure of T4519 is comprised of 6 anti-parallel β sheets at the N terminal domain and 9 α helices at the C terminal end. ATP and substrate binding region was present in-between N and C terminal domain. (Fig 6G). The overall acceptable quality and compatibility of the 3D atomic model was suggested by Ramachandran Plot (90.6% of the residues in the allowed regions), ‘Verify_3D’ (score 81.58) and ‘Errat’ (score 90.661) (S19A–B). After 5ns simulation, the resulted root mean square deviation (RMSD) plot showed that the 3D model was stable in aqueous medium at constant temperature and pressure (S19C Fig).

As per Mi Sun Jin.et. al. (2007), the tri-acylated lipopeptide induces the formation of an “m” shaped heterodimer of TLR1 and TLR2, but it was uncertain that how relatively large protein ligands interact with the TLR2/1 heterodimer. Subsequently, there were efforts where researchers had identified the critical region of TLR2 which interacted with other bacterial proteins like PPE18 and LT-IIb-B5 [53,54] lacking acyl chains. In their work, they had used different protein-protein docking programs to predict the most favorable binding site of TLR2 and afterwards, they had validated their result by site-directed mutagenesis.

Here we had used the similar approach to find out the most favorable site of the kinase binding to TLR2. We used ClusPro 2.0 (http://cluspro.bu.edu) protein-protein docking server for our prediction. The resulted 120 complexes were analysed to get the most favorable binding pose of T4519 with TLR2. In majority of these complexes (79 out of 120 conformations), the N terminal domain of the kinase bound to the border of the convex surface of the central domain of TLR2, which was similar to other TLR2 ligand proteins [53,54]. Further analysis of these 79 conformations revealed that 62 of the interactions involved glycine rich loop (12-GKGG-15) (Figs 6H and S19D) of the kinase, which is known to be involved in the interaction with other proteins [55]. Finally, to select the most favorable of the 62 interactions, we used three parameters, H-bond count, global energy value and electrostatic charge complementarity. The final selected model consisted of 30 H-bond interaction with TLR2, involving the residues from the lateral convex surface of the central domain and with a global energy value of -131.23, which was a measure of binding free energy of the complex. Interestingly, we found that the hydrophobic core between N and C terminal domain with the positively charged lining residues (K13,R90,H91,K125,R166,K207) and a deep cleft of negatively charged central residues (D123, D144, E77, E78) interacted mainly with the two loop regions (290–300 and 323–330) from the convex surface of TLR2 central domain that consists of positively charged hinge region (ARG321 at the tip of the loop), surrounded by negatively charged residues (D233,D235,D263,E264,D327) (Fig 6I and 6J). Moreover, these two loops from TLR2 provided a pedestal to the kinase hydrophobic surface (S19E Fig), giving rest to the complex. To confirm that the glycine-rich loop (12-GKGG-15) of T4519 binds to TLR2, we generated mutant *t4519* by site-directed mutagenesis of the glycine (G12/A, G14/A) and lysine (K13/V) residues and purified the recombinant T4519 and rSDM-T4519 proteins. TLR2 immunoprecipitated from HEK293 cells that expressed TLR2/6 and co-receptor molecules and were stimulated with rT4519 or rSDM-T4519 showed significantly less interactions with the latter (S19F Fig). Finally, to study if TLR2-T4519 interaction was functionally relevant for intracellular survival of *S*. Typhi, MoM cells were infected with Ty2, Ty2ΔT4519 and Ty2 expressing the mutant *t4519* gene (αSDM-T4519 Ty2). CFUs recovered after 24hrs were equal for both the mutant strains, but significantly less than the wild type bacteria. However, survival of all the above three strains was similar in TLR2 gene silenced TDM cells, suggesting that the glycine rich loop of T4519 was required to transduce survival signals through TLR2 (S19G–H).

### T4519 promotes intracellular survival through TLR2-Cystatin B-Cathepsin B-NFκB- ROS-LMP pathway

**Fig 7:**
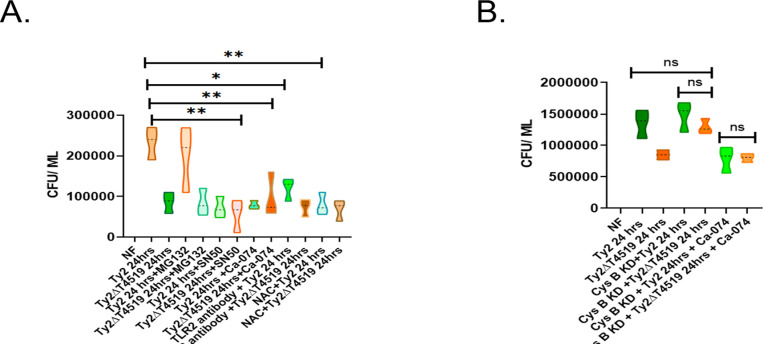
T4519 promotes intracellular survival through TLR2- Cystatin B- Cathepsin B- NF **κB- ROS- LMP pathway**. MoM (A) and TDM (B) cells were infected with wild type *S*. Typhi Ty2 or Ty2ΔT4519 for 30 minutes, followed by gentamycin protection assay as described under Materials and Methods. Cells were pre-treated with different inhibitors or subjected to cystatin B gene silencing as indicated. Intracellular CFU were counted 24hrs post-infection after culturing the bacteria overnight on LA plates. The above experiments were repeated three times and the values from all three experiments were plotted. NF, no Infection; ns, not significant. Statistical significance was calculated between the CFU recovered from the cells infected with Ty2 with and without the inhibitor/antibody pre-treatment. For TDM cells, statistical significance in bacterial counts were measured between Ty2- and Ty2ΔT4519-infected cells with and without cystatin B knockdown.

Since T4519 promoted survival of *S*. Typhi Ty2 in the MoM cells (S1 Fig), we investigated if the regulation of TLR2-cystatin B-cathepsin B-NF-κB axis by T4519 was responsible for enhanced bacterial survival. At the early hrs (0hr time point after infection), inhibitor treatment of PBMCs did not affect intracellular bacterial counts, indicating that phagocytosis of *S*. Typhi was not regulated by cathepsin B-NFκB pathways (S20 Fig). In contrast, significantly fewer live bacteria were recovered 24hrs post-infection from within the cells, which were pre-treated with the TLR2 antibody or the inhibitors of cathepsin B or NF-κB nuclear translocation. However, no changes in the intracellular CFU counts were found after pre-treatment with proteasome inhibitor, suggesting that cathepsin B-dependent, but proteasome-independent IκBα degradation and NF-κB nuclear translocation augmented T4519-induced survival of *S*. Typhi in the MoM cells as well as the TDM cells (Figs 7A and S20B). The mutant bacteria showed significantly reduced CFU counts than Ty2, both in the presence and absence of the inhibitors, suggesting the role of T4519 in TLR2-dependent activation of cathepsin B-NF-κB axis (Fig 7A). Scavenging of ROS at the beginning of infection abrogated T4519 induction within the bacteria, thereby compromising survival [44]. However, removal of ROS at a later time point (12hrs post-infection) after T4519 expression was induced also inhibited bacterial survival in MoM cells, suggesting that ROS mediated the T4519 survival signals (Figs 7A and S20B). As ROS generation by T4519 induces LMP, we checked the role of LMP in T4519-mediated survival of *S.* Typhi. Given that specific inhibitors of LMP are not commercially available, we induced LMP with LLOMe. We infected cells with Ty2 and Ty2Δt4519 followed by LLOMe treatment (0.5mM for 3hrs) at 12hrs PI. This led to LMP induction with identical intracellular CFU counts of both the strains at 24hrs post-infection (S21A and S21B Fig), suggesting that LMP induction by T4519 indeed promoted *S*. Typhi survival within MoM cells. Further, silencing of cystatin B gene augmented the survival of Ty2∆t4519 strain to the tune of the wild type bacteria, whereas prior inhibition of cathepsin B in cystatin B gene-silenced cells significantly reduced survival of both the strains (Fig 7B). While intracellular CFU for both the strains were several folds higher for the cell lines compared with the primary cells, as was also reported previously [56], significant difference between the counts of Ty2 and Ty2∆t4519 persisted in these cells too. Together the above results underscored the role of T4519 in promoting survival of *S*. Typhi in human macrophage cells through down-regulation of cystatin B upon engagement of TLR2, followed by the activation of cathepsin B-NF-κB axis and ROS generation, leading to LMP.

## Discussion

In this study, we have shown that eukaryote-like ser/thr kinase T4519 of *Salmonella* Typhi Ty2 promotes its survival in human monocyte-derived macrophage cells by inducing cytosolic cathepsin B-dependent activation of NF-κB, resulting in the surge of ROS generation within the cells, ROS-mediated LMP and loss of lysosomal staining. T4519 transcriptionally inhibits the cytosolic cathepsin B inhibitor, cystatin B through the activation of TLR2 signalling pathways, leading to residual active cytosolic cathepsin B, leaked from the lysosomes during the early hrs after the infection.

Most intracellular pathogens inhibit NF-κB signalling, either by acting upstream of IKKs or by directly targeting the core signalling components of the pathway. This prevents elicitation of a pro-inflammatory response, favoring bacterial survival and growth. Many prokaryotic ser/thr kinases were found to play a critical role in regulating this response, although an opposite function, leading to the activation of NF-κB and induction of pro-inflammatory cytokines and ROS was also observed for some eSTKs. *L. pneumophilia* LegK1 stimulates canonical NF-κB activation through direct phosphorylation and degradation of IκBα [35]. In addition, it also activates the non-canonical NF-κB pathway by targeting another IκB family inhibitor, p100 and processing it to generate p52 (NF-κB2). LegK1 functionally replaces host IKKs by targeting the same motif in the IκB family of proteins, but unlike IKKs, this function was independent of the upstream adaptor molecules (TRAF2, TRAF6) and kinases, (TAK1, NIK and MEKK3) [35]. NF-κB activation by LegK1 was reported to delay macrophage apoptosis, allowing more time for *Legionella* to replicate. The temporal dynamics of NF-κB response to *Bordetella pertussis* filamentous haemagglutinin (FHA) includes an early pathway activation, leading to pro-inflammatory cytokines production and inhibition of NF-κB upon longer exposure to FHA [57]. In contrast, obligate intracellular bacterial pathogen, *Rickettsia rickettsii* prolongs macrophage survival by inducing biphasic NF-κB activation in cultured human endothelial cells (ECs) – an early, transient phase at 3hrs and a late, sustained response from 18 to 24 hrs [58]. The early phase activation requires both IKKα and IKKβ, while the late response was IKKβ- and proteasome-independent and perhaps required a rickettsial or a host cell protease-mediated IκBα degradation [59,60,61]. Here we also report biphasic NF-κB activation in *S.* Typhi-infected MoM cells, where the early phase was proteasome-dependent, but independent of T4519 (Fig 1). In contrast, late NF-κB activation was induced by T4519 and mediated by proteasome-independent IκB degradation. A recent study suggested that multiple SPI-2 effectors of *S.* Typhimurium cooperate to inhibit NF-κB activation and their absence in *S.* Typhi, especially the lack of GogA and GtgA results in p65 phosphorylation in the infected THP-1 macrophages [62]. However, the above study did not specify the phosphorylation site(s) or the kinases involved in it as well as whether NF-κB phosphorylation followed proteasome-dependent or independent IκBα degradation. It is well established that NF-κB p65 gets phosphorylated at multiple residues, often by different upstream kinases after its release from the IκB complex. The most common phosphorylation sites on p65 are serine 276 and serine 536. PKA-C, MSK1, MSK2, Pim-1, RSK p90 and PKCα were reported to be responsible for serine 276 phosphorylation, while phosphorylation of serine 536 was mediated by IKKβ, RSK1, IKKα, IKKε and NAK/TBK1 [63]. Other reported studies identified deubiquitylation of IκBα by *S*. Typhimurium T3SS-2 effectors, AvrA and SseL being responsible for the inhibition of NF-κB activation [64,65].

Cathepsin B, a cysteine protease was implicated in the proteasome-independent, alternate pathway of NF-κB activation by several studies [45,66]. Following hypoxia/ischemia, NF-κB activation and pro-inflammatory cytokine and ROS induction in microglia/macrophages were mediated by cathepsin B-dependent autophagic degradation of IκBα that was unaffected by proteasome inhibitor. That IκBɑ is a substrate of cathepsin B was proved by the digestion of recombinant IκBα in an *in vitro* assay [66]. We also observed proteasome-independent, but cathepsin B-dependent IκBα degradation (Fig 2) and pro-inflammatory cytokine induction (Fig 4A, 4B, and 4C) in *S*. Typhi-infected MoM cells. A previous study suggested that cathepsin B promotes hepatic inflammation through NF-κB activation by proteolytic cleavage of Sirt1, a nuclear deacetylase that inhibits NF-κB transactivation capacity by deacetylating lysine310 residue [43]. However, Sirt1 played no role in cathepsin B-dependent NF-κB activation in *S*. Typhi infected cells in our study (S22 Fig).

We detected in the cytosol of MoM cells, infected with both Ty2 and Ty2Δt4519 enzymatically inactive cathepsin B (Fig 2G), which was subsequently activated only in the Ty2 infected cells. This correlated with T4519-dependent down-regulation of the default cathepsin B inhibitor, cystatin B expression (Fig 3A, 3B, and 3C). Infection of human oral epithelial cells with *P. gingivalis* or stimulation with bacterial LPS resulted in dose-dependent increase of cathepsin B and reduction in cystatin C expression levels. However, LPS effects were delayed compared with the whole bacteria, suggesting involvement of additional bacterial molecules [67]. In contrast, T4519 was solely responsible for cystatin B down-regulation (Fig 3A and 3B) and the activation of cathepsin B-NF-κB axis, promoting survival of *S.* Typhi in MoM cells (Fig 7). Other studies reported decreased interactions of cathepsin B with cystatin B and cystatin C in HIV-1 infected MoM cells, leading to increased Cathepsin B activity [68]. However, we found no changes incystatin C expression after Ty2 infection (S9A and S9B Fig), while we did not check cathepsin B interaction with the inhibitor cystatins. Cystatins also exert direct antimicrobial functions, such as those purified from chicken egg white. A20 peptide, mimicking the N-terminus of human cystatin C act against several pathogenic bacteria, including *E. coli*, *Acinetobacter* and MRSA [69,70]. However, antibacterial functions of cystatin B against *S.* Typhi in human macrophages were primarily mediated through the inhibition of cathepsin B (Fig 3C). This corroborates with the studies that reported increased resistance of mice and macrophages, lacking cathepsin B against *Francisella novicida* [71]. Also, macrophages lacking cathepsin B do not support productive intracellular replication of *L. pneumophila* harboring wild type RpsL, which induces cell death via LMP [72]. Cathepsin B functions as a negative regulator of lysosomal biogenesis and autophagy through mTor-mediated cleavage of lysosomal Ca++ channel TRPML1, suppressing TEFB [71].

Recent studies suggested that intracellular persistence and growth of *S*. Typhi is promoted by NF-κB activation that prevents death of infected macrophages [62]. This observation corroborates with another recent report that documented M1 macrophages to house the replicating *S*. Typhi, since NF-κB polarizes macrophages to M1 phenotype and remains the major transcription factor in these cells [48]. However, NF-κB, at the same time is a key molecule that orchestrate the antibacterial immune response of macrophages. It is currently unknown if *S*. Typhi could directly exploit NF-κB signalling pathways to boost its intracellular survival. We found here that *S.* Typhi induced LMP through Cystatin B-Cathepsin B-NF-κB pathways in a T4519-dependent way, leading to reduced number of acidic vesicles in the infected cells and increase in intracellular pH (S12 Fig). However, lysosome dependent cell death did not follow LMP in our study, perhaps because of the activation of anti-apoptotic pathways by NF-κB.

Lysosomal hydrolases are powerful bactericidal agents and hence, LMP leading to loss of lysosomal enzymes has remained an important immune evasion mechanism for bacterial pathogens. *B. anthracis* lethal toxin induces LMP through activation of NLR signalling, leading to apoptosis [73]. On the other hand, pneumolysin selectively induces loss of lysosomal acids (LLA)/ LMP, leaking smaller molecules (up to 40 kDa) into the cytosol. This is mediated through multiple TLR activation, especially TLR2 and TLR4, but independent of the pore-forming ability of pneumolysin and does not induce cell death [27]. In contrast, T4519 exclusively used TLR2 to induce LMP, leading to marked loss of lysosomal staining and increased bacterial survival (Figs 6F and 7). TLR-mediated augmentation of phagosomal persistence was also reported for *S*. Typhimurium, where TLR2, 4 and 9 together was found to provide cues to the bacteria to sense the phagosome and regulate virulence genes required for intracellular survival and replication [74]. *Brucella suis* LPS, a ligand for TLR4 may inhibit phagosome-lysosome fusion [75]. To the best of our knowledge, this is the first study that demonstrated a direct role of NF-κB in promoting phagosomal survival of intracellular bacteria and inducing LMP.

ROS production by macrophage helps to clear bacterial infection, but excessive ROS generation, especially within lysosomes can trigger LMP through multiple mechanisms, including lysosomal free iron-dependent H_2_O_2_ and hydroxyl radical generation, membrane phospholipase A_2_ degradation etc [29]. ROS and NF-κB have reciprocal regulation where one can induce the other [76]. NF-κB induces ROS by activating NADPH oxidase, NOX2 and up-regulation of iNOS [76]. We observed a surge in ROS production in Ty2-infected macrophages by a novel, T4519-activated Cathepsin B-NF-κB pathway (Fig 4D and 4E) and ROS mediated LMP (Fig 5F), promoting phagosomal persistence of *S.* Typhi.

Reactive nitrogen species (RNS) include nitric oxide (NO)-derived compounds, such as nitroxyl anion (NO^-^), nitrate (NO3^-^) and its structural isomer peroxynitrite (ONOO^-^), nitrosonium cation (NO^+^), higher oxides of nitrogen, S-nitrosothiols, and dinitrosyl iron complexes. Pathogen-associated molecular patterns (PAMPs) and the type III secretion system 1 (T3SS-1) effectors from *Salmonella enterica* augment transcription of inducible NO synthase (iNOS or NOS-2) in the macrophages. NO and its derivatives exert bacteriostatic or bactericidal functions by targeting the electron transport chain, metabolic enzymes, transcription factors and DNA and DNA-associated proteins, besides modulating the expression of bacterial virulence genes encoded by T3SS-2 and host immune responses. NO⋅ targeting of lipoamide dehydrogenase (LpdA) activity results in methionine and lysine auxotrophy due to reduced succinyl-CoA availability [77].). However, *S.* enterica, which can generate ATP via both oxidative and substrate-level phosphorylation, are less susceptible than other intracellular bacteria to the antimicrobial actions of NO and employs multiple mechanisms against it, including down-regulation of NOS2 expression via RNA interference [78], interupting trafficking of NOS2-containing vacuoles [79] and adaptive detoxification of NO by derepression of *hmp* gene [80]. High-affinity ZnuABC zinc uptake system promotes *Salmonella* pathogenesis during the nitrosative stress[81], while ZntB and ZitB zinc efflux systems protect *Salmonella* from nitrosative stress [82]. Intriguingly, *Salmonella* migrates towards nitrates produced in the lamina propria by host phagocytes, promoting its invasion of Peyer’s Patches [83]. Host NO elicits early *S*. Typhimurium replication in the mouse macrophages by activating *Salmonella* pathogenicity island-2 (SPI-2) genes through Fnr and PhoP/Q two-component system [84]

Toll-like receptors recognize pathogen-associated molecular patterns to promote innate immunity. In general, TLR2 plays a more important role for protection against Gram positive bacteria, while TLR4 for the Gram negative species. However, several bacterial pathogens have evolved mechanisms to exploit TLR signals to augment their virulence. *Yersinia* spp. LcrV induces TLR2-mediated immunosuppression and a point mutation in the TLR2-binding epitope of LcrV abrogates interactions and decreases virulence [85]. *S*. aureus prolongs its own survival within the phagosomes of macrophages by inhibiting the generation of superoxides other than NO through TLR2-dependent JNK activation [86]. While TLR2 plays a critical role in the early stage macrophage defence against *Mycobacterium tuberculosis* by suppressing proliferation, by direct bactericidal effects or induction of apoptosis, it may help Mtb in the later stages to escape recognition by inhibiting MHC II expression or inducing IL-10 and IL-4 using the bacterial 19 kDa glycolipoprotein (p19), which is both cell wall-associated and secreted and a candidate virulence factor [87]. We show here that T4519 signals through TLR2 to suppress cystatin B expression, allowing enzymatically active cytosolic cathepsin B to induce LMP by the activation of NF-κB-ROS pathway. *Clostridium* difficile toxin (CDT) activates TLR2 and induces robust inflammation and tissue damage by activating NF-κB, thereby promoting bacterial pathogenesis [88]. On the other hand, *T. denticola* contains a heat-labile inhibitor of TLR2 that suppresses human beta densin 3 (HBD-3), a cationic antimicrobial peptide in the gingival epithelial cells [89]. TLR2 knockout mice have decreased pathology and increased survival compared with the wild-type mice from sepsis caused by *Burkholderia pseudomellei*, suggesting that TLR2 mediates morbidity and mortality in murine models of this pathogen [90].

In conclusion, we report here a novel mechanism of cytosolic cathepsin B-mediated NF-κB activation by *S.* Typhi that promotes bacterial survival within human macrophages. T4519, a bacterial eSTK indirectly activated cathepsin B by down-regulating the expression of its inhibitor molecule, cystatin B in a TLR2-dependent way. This led to a surge of ROS production by NF-κB, leading to ROS-induced LMP.

There are few limitations of the study. We did not directly demonstrate T4519 and TLR2 interaction in the infected macrophages. Instead, we had shown this interaction by co-immunoprecipitation experiment in TLR2 over-expressed 293 cells and purified T4519 protein. The absence of significant cell death despite robust LMP induction in *S.* Typhi infected macrophages could perhaps be due to the simultaneous activation of anti-apoptotic signals by NF-κB, although we have not studied this aspect.

## Materials and methods

### Cells and reagents

THP-1 cell line (cat # TIB-202.) was bought from American Type Culture Collection (ATCC). RPMI 1640 (cat # 31800022) and FBS (cat 26140079) were bought from GIBCO. Bacterial culture media, such as Luria broth (cat # 241420) and Luria agar (cat #211829) were procured from BD Difco. RNA was isolated using TRISOL reagent (cat #T9424) from Sigma. cDNA kit reagents were purchased from Bio-Rad (cat #1708891) and SYBR green Master Mix from ABI (cat # 4368708.). TNF-α and IL-6 ELISA kits were purchased from R&D systems (cat # DTA00D for TNF-α and D6050B for IL-6). All cell culture plastic ware used were from Nunc. Following antibodies were procured from Cell Signalling Technology: IκBα (cat # 9242S) p65 (cat # 8242S), histone H4 (cat # 13919S), β-actin (cat # 3700S), m-TOR (cat # 2983S), galectin-1 (cat # 12936S), TLR9 (cat #.13674S) and HRP-conjugated anti-rabbit IgG (7074P2). Cystatin B (cat # PA5110828) and cystatin C (cat # PA5110779) were bought from Thermo Fisher Scientific, while antibodies against cathepsin B (cat # ab214428), cathepsin B substrate Magic red (cat # ab270772), TLR2 (cat #ab213676) and TLR4 (cat # ab 13556) were from ABCAM. Horseradish peroxidase (HRP)-conjugated anti-mouse IgG were bought from Pierce. Other reagents, such as MG-132 (cat #474790.), SN-50 (cat #.SML1471), Ca-074 (cat #C5732), Triton X-100 (cat #T8787), Monensin (cat # M5273) and Hoechst (cat #94403) were bought from Sigma. Reagents for confocal microscopy such as Dextran Alexa-Fluor 546 ((cat # D22911), fluorochrome (Alexa-Fluor)-conjugated secondary antibodies (anti-rabbit 594 IgG - cat #.A11012., anti-mouse 546 IgG - (cat #.A-21123 and anti-rabbit 633 IgG - cat #..A21070.) were taken from Invitrogen. Cystatin B knock out si-RNAs and transfection reagents were procured from Santa Cruz Biotechnology. H2DCFDA (cat # DCF D399) and LysoSensorYellow/Blue DND-160 (cat # L7545) were purchased from Invitrogen. siRNAs for p65) were procured from Ambion. All other reagents were bought from Sigma, if not otherwise mentioned.

### Bacterial strains, plasmids and primers

Bacterial strains used include *Salmonella* Typhi Ty2 (cat #700931 ATCC), *Salmonella* serovar Typhimurim LT2 (LT2) (cat # 14028s ATCC) and the laboratory generated strains, such as *Salmonella* Typhi Ty2ΔT4519 and α-Ty2ΔT4519, Ty2αSDM-T4519 [44]. pAKGFP1 plasmid (cat # 14076) was from Addgene. Cystatin B siRNA (cat # sc-4443), siRNA Dilution Buffer (cat # sc-29527), siRNA Transfection Reagent (cat # sc-29528), siRNA Transfection Medium (cat # sc-36868), siRNA Reagent System (cat # sc-45064) and control siRNA-A (cat # sc-37007) were purchased from Santa Cruz Biotechnology. List of primers used in this study was incorporated in S1 Table.

### Cell culture

THP-1 cell line was cultured in the complete RMPI medium (RPMI 1640, supplemented with 10% fetal bovine serum (FBS) and Penicillin-Streptomycin (Gibco)). Cells were differentiated into macrophages by adding 100 nM of phorbol 12-myristate 13-acetate (PMA) for 24 h, followed by maintaining the cells in the culture media in the absence of PMA for 24 hrs before the experiment. 100 nM PMA did not induce M1 cytokines surge in non-infected TDM cells, was proved earlier [44]. HEK293 cells were grown in DMEM containing 10% FBS.

Peripheral blood mono-nuclear cells (PBMC) were isolated from the human whole blood, provided by the Blood Bank of Apollo Glenicals Hospital, Kolkata. Blood was diluted at 1:1 ratio with 1X PBS and loaded onto Histopaque (Sigma) at a ratio of 1 volume Histopaque and 3 volume of diluted blood in a 15 ml polystyrene tube. The tube was centrifuged at 550 xg for 30 minutes with break. PBMCs separated as a while ring was carefully aspirated and washed with 1X PBS (HiMedia). After removing the contaminating RBCs using the RBC lysis buffer, cells were counted using a Neubauer Chamber and seeded at a density of 2* 10^6 per well in a 24 well plate in RPMI medium supplemented with 10% male human serum. (Sigma, cat # H4522.). After 24 hrs, media was replaced with fresh RMPI 1640, containing 50 µM rh-MCSF (R & D, cat # 216MC025CF.), which was repeated on alternative days till day 7. Cells were viewed under microscope to confirm monocyte to macrophage conversion.

### RNA interference

PMA-differentiated THP-1 cells were transfected with the control or gene-specific siRNA, using siPort as per the manufacturer's instructions (Invitrogen). Briefly, siRNA complex and siRNA transfection reagent were diluted in the siRNA transfection medium and incubated at the room temperature for 30 minutes. Cells were overlaid with the transfection mixture for five hrs at 37°C in the presence of 5% CO_2_, followed by culture in complete RPMI 1640 medium. Gene knockdown was confirmed by immunoblots 48 hrs post-transfection. For cystatin B knockdown, cystatin B siRNA and Transfection medium were mixed to make solution A and transfection medium and transfection reagent were mixed to make solution B. THP-1 cell-derived macrophages were incubated with the mixture of solution A and B for 6 hrs. After 24 hrs, the culture media was replaced with fresh media and 48 hrs later, the cells were lysed by RIPA buffer and western blotting were done using cystatin B antibody to confirm gene knockdown.

### Gentamycin protection assay

Gentamicin protection assay was done with both THP-1- and MoM-derived macrophages. THP-1 macrophages were infected at a multiplicity of infection (MoI) of 50 for Ty2 and LT2 and 100 for Ty2Δt4519. The corresponding MoI for MoM cells were 20 and 40, respectively. Prior to infection, bacterial cultures were opsonized with complete RPMI1640. Cells were synchronized with the opsonized bacteria by centrifugation at 400 × g for 5 min, followed by incubation for 30 min at 37°C (5% CO2). Extracellular bacteria were removed by washing the cells for 3 times with 1× phosphate-buffered saline (PBS) and the infected cells were cultured in complete RPMI medium, containing 100 μg/ml of gentamicin (Gibco) for 1 hr, followed by 15 μg/ml of gentamicin till the end of the experiment. Cells were lysed with 0.25% Triton X-100, dissolved in 1xPBS. Intracellular CFU were counted after culturing the bacteria overnight on LA plates at 37 º C.

### Cell viability assay

MoM cells were seeded at a density of 5 × 10^4 cells/well in 96-well plates and infected with bacteria as described in the “Gentamicin protection assay” section. The release of lactate dehydrogenase (LDH) into the culture supernatants was quantified using the In vitro Toxicology LDH assay kit (Sigma-Aldrich) following the manufacturer's instructions.

### ELISA

Supernatants from non-infected and infected cells were taken at desired time-points and TNF-α,IL-6 in the culture supernatants were measured by TNF-α,IL-6 ELISA kit (R&D) by following the manufacturer's protocol.

### Western blot

At the end of the experiments, cells were lysed with ice cold RIPA Buffer (50 mM Tris HCl, 150 mM NaCl, 1.0% NP-40, 1.0 mM EDTA, 0.1% (w/v) SDS, 0.01% sodium Azide, 0.5% Sodium Deoxycholate, pH 7.3), containing protease inhibitor cocktail. Protein concentrations the lysates were measured by Bradford Assay (Sigma). Equal concentrations of total proteins from different cell lysates were run in SDS-PAGE and transferred to a PVDF membrane (Millipore). Blots were blocked with TBS-T, containing 5% BSA (Sigma) for 1 hr, followed by overnight incubation at 4°C with primary antibodies, dissolved in the blocking buffer. Next day, blots were washed for 3 times with TBS-T and probed for 1 hr at room temperature with HRP-conjugated secondary antibodies, diluted (1:5000) in TBS-T containing 3% BSA. Blots were washed 3 times with TBS-T in an orbital shaker and developed with the substrate Super Signal West Pico (Thermo Fisher). Chemiluminescence signals were captured in ChemiDoc MP imaging system (Bio Rad).

### Co-immunoprecipitation

HEK293 cells expressing TLR2 were seeded (1 x10^8^/ cells per well in a 6 well plate) and treated either with 3 µg of rT4519 or rSDM-T4519 proteins for 24 hrs. Cells were washed with ice-cold PBS and cross-linked with 1% para-formaldehyde (PFA) for 10 minutes, followed by two washes with ice-cold PBS to remove PFA. Cells were lysed with Nonidet P-40 lysis buffer containing protease inhibitors, the lysates were cleared by centrifugation at 14,000 гpm for 20 minutes at 4 °C and the supernatants were collected in fresh tubes. After determination of the protein concentration by Bradford Assay, primary antibodies (1:50 dilution) were added to the cell lysates (containing 600 µg of total protein in each tube) and the mixtures were kept overnight on a rocker at 4 °C. 50 µl of protein A/G agarose slurry (50%) (Santa Cruz) equilibrated with the lysis buffer was added to each cell lysate and incubated for 4 hrs on a rocker at 4 °C. Protein A/G agarose beads were collected by pulse centrifugation (14,000 rpm for 5 seconds). Supernatants were removed and the beads were washed three times with 500 µl of cold PBS. Agarose beads were resuspended in 1X sample loading buffer and boiled at 94 °C for 10 minutes to dissociate the bound proteins. Proteins were resolved in SDS-PAGE and probed with monoclonal anti-His or TLR2 antibody in a western blot assay.

### Extraction of nuclear proteins

Cells were treated with cytoplasmic extraction buffer (tris HCL- PH 8.0 10 Mm sucrose, 0.34 M calcium chloride, 3 mM magnesium chloride, 2 Mm EDTA, 0.1 Mm DDT, 1 Mm NP40 0.5%, protease inhibitor 1 tablet for 10 ml, phosphatase inhibitor 1 mM) on ice for 10 min and harvested by centrifugation at 3500 g for 15 mins at 4 ºC. Nuclear proteins were extracted from the cell pellet by treating with the nucleus extraction buffer (HEPES buffer 20 mM, EDTA 3 mM, Glycerol 10%, potassium acetate 150 mM, magnesium chloride 1.5 mM, DTT 1 mM, NP40 0.5% protease inhibitor 1 tablet for 10 ml, phosphatase inhibitor 1 mM) for 10 mins on ice. The mixture was centrifuged again at 14000 xg for 15 mins at 4 ºC. The supernatants containing the nuclear proteins were analysed by western blots.

### Cytoplasmic and lysosomal fractions isolation

Lysosomal membrane fractions (LF) and cytosolic fractions (CF) were prepared as described before [91,92]. Briefly, cells were washed with chilled 1X PBS, gently scrapped and centrifuged at 600x g for 5 minutes at 4 º C. Cells were incubated with twice the volume of MSH buffer (200 mM mannitol, 70 mM sucrose, 20 mM HEPES (pH 7.5), 1mM EDTA, 100 µM PMSF) for 45 minutes on ice, followed by lysis using a 25 G needle until 75% of the cells were trypan blue positive. Cells were centrifuged for 5 minutes at 350x g to pellet the cellular debris and the nuclei. The supernatant was centrifuged to 100000 X g for 1 hr to obtain the cytosol. The pellets found here contains lysosomal fraction. To obtain the crude membrane/ lysosomal fraction, (containing all other organelles except nuclei), the pellets were resuspended in MSH buffer containing 1% Triton-X.

### Confocal microscopy

For confocal microscopy experiments, coverslips were coated with rat tail collagen for 6 hrs at 37 º C in 24 well plates. After drying, cells were seeded onto the coated cover slips. On the day of infection, each well of a 24 well plate contained 2*10^5 PBMC-derived macrophages or 5*10^5 THP-1 cells. For antibody staining, the infected cells were fixed with 4% PFA in PBS for 15 min at 37 º C, permeabilised with 0.1% Triton X-100 in 1xPBS for 10 min, followed by blocking with 1% BSA in the same buffer for 30 min. Cells were incubated overnight at 4 ºC with the primary antibodies diluted in PBS containing 1% BSA (1:5000 ratio). Fluorochrome-conjugated secondary antibodies were diluted in the same buffer (1:10000) and incubated for 1 hr at room temperature in the dark. Nucleus was stained by incubating the cells with Hoechst dye (Working concentration 10 µg/ml in PBS) for 30 min in the dark. Lysosomes were stained with 1 µM Lysotracker DND88 for 1 hr at 37ºC. For Dextran Alexa-fluor staining, cells were incubated overnight with Dextran Alexa-fluor in complete cell culture medium to allow dextran to reach the vacuoles of the cells including the lysosomes. In the acidic vacuoles, the dye produces red fluorescence. For Magic Red staining, the infected cells were incubated in Magic Red reagent 1:5000 dilution for 1 hr at 37º C. Quantification under confocal microscope was done by counting 50 cells from the random fields from each experimental sample. Puncta were counted using the “particle analysis” mode of Fiji ImageJ software. Size of the puncta for every experiment was kept the same and the background noise considered as puncta by the software was eliminated manually Colocalization was measured by using the ZEISS software after selecting the fields individually.. All the images were captured by an Axio Observer LSM710 ZEISS confocal microscope, using 63X/1.4 oil immersion lens at the scale bar of 5 µm. The confocal images were processed by ZEN-Blue 3.1 software.

### Transmission electron microscopy

MoM cells seeded in 6-well plates (2x10 6 cells/well) were pulse-chased with 25 nm BSA-gold tracer (Electron Microscopy Sciences) for 4h. Cells were washed with PBS and infected as described under ‘gentamicin protection assay’. Cells were fixed with phosphate buffer (pH 7.4) containing 3% glutaraldehyde and post-fixed in 1% osmium tetra oxide, followed by dehydration using ascending acetone series and embedding in Agar100 resin (Agar Scientific). Ultrathin sections were examined using a FEI Tecnai 12 BioTwin Transmission electron microscope operated at 100 kV.

### Gene expression analysis

Twenty-four well plates were seeded with 2*10^2 PBMC derived macrophages or 5*10^5 macrophages generated from THP-1 cells. After infection, total RNA was isolated using the TRIzol reagent (Invitrogen) following the manufacturer's protocol. 1 µg of host mRNA was used for cDNA preparation using SuperScript II reverse transcriptase. Quantitative PCR (qPCR) was done in a StepOnePlus system (ABI) by SYBR green master mix. Relative quantitation was done by the comparative threshold cycle (CT) method [93]. The change of expression of the desired gene was normalized against the β-actin gene by applying the formula 2ˉΔΔCT, where -ΔΔCT is calculated as ΔCT (sample) - ΔCT (calibrator), and ΔCT is the CT of the target gene subtracted from the CT of the housekeeping gene (β-actin). The calibrators used in our experiments were uninfected PBMC derived macrophage cells.

### Measurement of ROS

To measure ROS generation, PBMC derived macrophage cells were washed twice with PBS after infection at the indicated time points, and incubated with PBS containing10 µM of CM-H₂DCFDA (Invitrogen) at room temperature for 45 minutes. The dye was removed by washing with PBS and the cells were re-suspended in complete RPMI 1640 medium to determine the ROS levels by flow cytometer (FACS Arya II, BD), using excitation sources and filters appropriate for FITC after gating on the basis of the basal levels of fluorescence intensity for unstained cells.

### Measurement of vacuolar pH

For vacuolar pH measurement, buffers of different pH were made to obtain a standard curve by following the protocol described previously [50]. (S2 Table). Two days before the experiment, THP-1 cells were seeded in presence of PMA onto 24 well plate at a density of 5 × 10^5^/ well. On the day of the experiment, the cells were incubated at 37°C in 1 mL of pre-warmed, complete RPMI medium, containing LysoSensor Yellow/Blue DND-160 (1 μM) for 5 min followed by rinsing with 1X PBS. For standard curve preparation, each well of cells was rinsed once with pH calibration curve buffers. For each well, a different pH calibration buffer is used from pH 7.5 to 3 and incubated for 5 min in 500 μL of the same buffer. The fluorescence intensity measurement data for the microplate readings at both wavelengths (e.g., 440 and 540 nm) were exported to Microsoft Excel and the intensity ratios of the shorter and longer wavelengths for each pH calibration curve buffer were calculated. The average fluorescence intensity ratios were plotted to generate a pH calibration curve. After completion of the gentamycin protection assay, the infected and uninfected wells were incubated with lysosensor dye for 5 min, rinsed with 1x PBS and the reading was taken using a fluorimeter at both wavelengths (e.g., 440 and 540 nm).. The intra-vacuolar pH of the infected cells were determined by plotting the values on the standard curve.

### Homology modelling

T4519 sequence from *Salmonella* Typhi Ty2 strain was subjected to HHPRED [94] and PSIPRED v3.0 (http://bioinf.cs.ucl.ac.uk/psipred/) [95,96] server to predict the secondary and tertiary structures. Fold prediction analyses and psiBLAST [97] against protein data bank (PDB) sequences suggested strong structural similarity between T4519 and the ser/ thr kinase family. The crystal structure of PknB from *Mycobacterium tuberculosis* (PDB ID 1O6Y), which possess maximum sequence identity (29%) with T4519 was used as a template to model the three-dimensional (3D) structure of T4519. The model was generated using Modeller 9v7 [98] and further refined with repetitive loop modelling [99]. Finally, the potential energy of the model was minimized using steepest descent, followed by conjugate gradient method in Discovery Studio 2.5. Three dimensional models were filtered based on the DOPE score and validated using PROCHECK [100] and verify_3D [101,102] structure validation tools. To check the stability of the T4519 atomic model, a short duration (5ns) molecular dynamic simulation in GROMACS 4.**5** was performed [103].

### Protein-protein interaction study

To check the interaction between T4519 and TLR2, we retrieved the TLR2 crystal structure from PDB (PDB ID 2Z7X), which is a TLR1-TLR2 heterodimer [104] and docked it with T4519 using ClusPro 2.0 protein-protein docking server [105] with all the default parameters. The docked complexes were refined further in FireDock server (http://bioinfo3d.cs.tau.ac.il/FireDock/) and the global energies of the complexes were measured.

### Statistical analysis

All the graphs were plotted and the statistical analysis was done by unpaired Students t-test in GraphPad Prism Version 8.0.1 with P <0.05 considered as significant.

## Supporting information

S1 Fig Intracellular bacterial CFU counts in MoM cells. MoM cells were infected with wild type *S.* Typhi or Ty2ΔT4519 for 30 mins followed by gentamycin protection assay as described under Materials and Methods. CFU determined T4519 mediated survival. Intracellular CFU were counted after 24 hrs of infection following cell lysis and the lysates were platted on LA plates, which were incubated overnight at 37° C. Here P*< 0.05 compared between CFU of Ty2 24 hrs and ΔT4519Ty2 24 hrs.
(PDF)

S2 Fig LDH was measured in the culture supernatants of the cells. MoM cells were infected and LDH was measured from the culture supernatants of the cells at 24 hrs P.I.
(PDF)

S3 Fig CFU were calculated at different MoI, 24 hrs after infection in MoM cells. Here P*< 0.05 compared between CFU of Ty2 20 MoI and ΔT4519Ty2 20 MoI. Here P*<0.05 compared between CFU of Ty2 20 MoI and ΔT4519Ty2 10 MoI. Significance was calculated using two tailed unpaired T test. Statistical analysis was done by using GraphPad Prism 8. NS stands for not significant.
(PDF)

S4 Fig Delayed p65 nuclear translocation is T4519 dependent and early p65 nuclear translocation is proteasome dependent in human MoM cells. MoM cells were infected with wild type *S*. Typhi or Ty2ΔT4519 for 30 mins followed by gentamycin protection assay as described under Materials and Methods. A. Quantification of IκB-α and β-actin western blots. Quantification was done by using ImageJ software and graph is plotted in graph pad prism 8.B-D–p65 confocal microscopy staining after infection with GFP tagged bacteria. MoM cells were incubated with indicated inhibitors followed by infection. Alexa-fluor conjugated secondary antibody (Alexa-fluor 594 anti rabbit) was used for p65 staining and nucleus was stained by Hoechst (blue). Here NF stands for no Infection. The above experiments were repeated three times and the values from those three experiments were plotted. Error bars represent SD. Significance was calculated using two tailed unpaired T test. Statistical analysis was done by using GraphPad Prism 8. NS stands for not significant. A representative image from each experiment was given. E.Quantification of fluorescence colocalization of Hoechst (blue) and p65 (red), done by taking random fields, from which in a total of 50 cells, blue and red co-localization were measured by using LSM 710 Zeiss Zen Blue software of confocal microscope. P***< 0.001,compared between Ty2 1 hr and MG132 Ty2 1 hr infected cells and P***< 0.001, compared between Ty2ΔT4519 Ty2 1 hr and MG132 Ty2ΔT4519 1 hr infected cells.
(PDF)

S5 Fig Delayed, but not early p65 nuclear translocation in S. Typhi-infected human MoM cells are cathepsin B dependent. MoM cells were incubated with indicated inhibitors followed by infection. A. Quantification of IκB-α and β-actin western blots. Quantification was done by using ImageJ software and graph is plotted in graph pad prism 8. B-D. p65 confocal microscopy staining was done. Alexa-fluor conjugated secondary antibody (Alexa-fluor 594 anti rabbit) was used for p65 staining and nucleus was stained by Hoechst (blue).
(PDF)

S6 Fig Both Ty2 and Ty2ΔT4519 infected MoM cells showed cytosolic Cathepsin B between 8–12 hrs PI. Infected MoM cells were lysed and cytoplasmic fraction (CF) and lysosomal fraction (LF) were isolated. Western blot was done by cathepsin B antibody at 8 hrs, 12 hrs and 24 hrs PI respectively. Tubulin was used as loading control.
(PDF)

S7 Fig Ty2 infected MoM cells showed active cytosolic cathepsin B at 12 hrs PI. MoM cells were infected with GFP tagged bacteria and stained with magic red. Nucleus is stained with Hoechst. Representative images from each experiment was given. Images were taken in Zeiss LSM 710 confocal microscope.
(PDF)

S8 Fig Expression level of Cystatin B remained unchanged after LT2 infection. m-RNA fold changes of cystatin B measured by RT-Qpcr. β-actin was used as control gene.
(PDF)

S9 Fig Expression level of Cystatin C remained unchanged after Ty2 infection. (A). m-RNA fold change of cystatin C was measured by RT-PCR. β-actin was used as control gene. (B). The western blot of cystatin C. The time points taken was 12 hrs PI. β- actin was used as loading control for all the blots.
(PDF)

S10 Fig Expression of Cystatin B after si-RNA KD. WB showing expression of cystatin B in Scramble Control and knockout cells.
(PDF)

S11 Fig NF-κB activation by Cys B and Cat B regulated ROS. A,C,D. MoM cells were infected followed by staining with PBS containing 10 µM of CM-H₂DCFDA (Invitrogen) and FACS was done in FITC filter. NF stands for No Infection. B. ELISA to quantitate IL-10 concentration in MOM culture.C-D. Representative histogram images of FACS.The shift of MFI (Mean Fluorescent Intensity) of FITC peak was shown by an arrow. E. Nuclear translocation of p65 was hindered by SN-50 treatment proved by confocal microscopy.
(PDF)

S12 Fig Ty2 infection increased vacuolar pH. (A). MoM cells were stained with lysosensor yellow blue DND dye and exposed to different pH containing buffer from 3 to 8. Excitation and emission ratio of 440/540 was taken in a fluorimeter. The values were plotted to make the standard curve. (B). MoM cells were infected and then incubated in lysosensor yellow blue DND dye and excitation and emission ratio of 440/540 was taken in a fluorimeter. The value was plotted in the standard curve to obtain the correct ph. Graphical representation of different pH was given.
(PDF)

S13 Fig Cathepsin B-NF-κB induces LMP at later time point of infection proved by Galectin-1 puncta assay. (A-F). MoM cells were infected and stained with Galectin 1 antibody followed by Alexa-fluor conjugated secondary antibody and nucleus was stained by Hoechst (blue). (C). Quantification of Galectin-1 puncta was done by Fiji ImageJ software “particle analysis” tool. Non-punctas were eliminated manually. Cells were randomly selected from each field. N=50 cells., under LSM 710 ZEISS confocal microscope. A representative image from all experiments was given.
(PDF)

S14 Fig T4519 induces LMP through Cathepsin B-NF-κB. (A-E). Cells were incubated with Dextran Alexa-fluor containing media overnight and then infected with GFP tagged bacteria. (E). Quantification of dextran puncta. N=50 Cells (F). Lysotracker red staining after infecting p65 si-RNA KD TDM cells. TDM cells were treated with p65 si-RNA to knock down p65 followed by infection.
(PDF)

S15 Fig Cystatin B-Cathepsin B-NF-κB induces LMP. (A-D). Cystatin B KD was done by si-RNA. (A-B). Cystatin B KD TDM cells were infected and stained with Galectin 1 antibody followed by Alexa-fluor conjugated secondary antibody (C). THP-1 derived macrophages were incubated with Dextran Alexa-fluor containing media overnight and then infected with bacteria. (D). Quantification of dextran puncta was done by Fiji ImageJ software “particle analysis” tool. Non-punctas were eliminated manually. Cells were randomly selected from each field. N= 50 cells. Representative images from all these experiments were given. Confocal images were taken in LSM 710 confocal microscope and statistical analysis were done in Graph Pad-Prism 8 by unpaired Students’ T-test.
(PDF)

S16 Fig TLR2 played a critical role for Ty2 survival. (A). CFU of wild type bacteria after infecting TDM cells at 24 hrs. TDM cells were treated with different si-RNA of that particular TLR to knock down. Knock down was tested by western blot. Tubulin was used as loading control. (B). CFU of wild type and mutant bacteria at 24 hrs PI after lysing MoM cells. Error bars, means, standard deviations were done for three independent experiments, *** by students’ T Test. All statistical analysis was done in Graph Pad-Prism 8.
(PDF)

S17 Fig YB-1 m-RNA expression is regulated by TLR-2. (A-B). m-RNA expression was measured by RT-PCR after infecting the MoM cells with Ty2. (A). m-RNA fold change of NOVA-1, (B). m-RNA fold change of YB-1. Error bars, means, standard deviations were done for three independent experiments, *** by students’ T Test. All statistical analysis was done in Graph Pad-Prism 8.
(PDF)

S18 Fig TLR2 KD terminated LMP. Lysotracker red staining after 24 hrs of infection in TDM cells. TDM cells were treated with TLR2 si-RNA to knock down TLR-2 before infection with GFP tagged bacteria. A representative image of all experiments were given. Image captured in Zeiss LSM 710 confocal microscope.
(PDF)

S19 Fig T4519 functions as TLR2 ligand. (A-E). Homology modeling of T4519. (A). Ramachandran plot of the 3D model structure of T4519. Ramachandran plot for the t4519 model structure shows 90.6% of the residues are in the allowed regions whereas 8.6% and 0.9% residues are observed in the additional and generously allowed regions, respectively (B). ERRAT plot shows the statistics of non-bonded interactions between different atoms. The overall quality of the modeled protein structure is indicated by the score of 90.661 (Low resolution structures produce values around 91). (C). 5 nanoseconds molecular dynamics simulation plot of T4519 showing relative deviation of the protein backbone atoms during simulation. Polynomial curve (Red) shows that the protein is quite stable after 2 ns as it fluctuates within the range of 0.4-0.5 nm. D. 62 interactions between TLR2 and T4519 produced by ClusPro 2.0 docking server. Figure represents favored interactions, which include 11 Vanderwalls, 9 Hydrophobic, 22 Electrostatic and 20 balanced interactions. E. The cartoon representation of t4519 and TLR2 complex. The image showing two loops from TLR2 (in CPK) positioned between N and C terminal hydrophobic region of t4519. F. Stable expression TLR2/6 HEK293 cells were transiently transfected with TLR accessory proteins. Cells were stimulated with rT4519, rT4519-SDM (G12A/K13V/G14A) for 24 hrs and immunoprecipitation were done. (G-H). Cells were infected with the indicated bacterial strains. CFU counts of intracellular bacteria recovered from the lysed cells after 24 hrs were plotted.
(PDF)

S20 Fig A. Phagocytosis of S. Typhi was not regulated by catB-NFκB pathways. Bacterial CFU at 0 hr time point after infection shows no significant difference. MoM cells were treated with Ca-074 (30 µM), SN50 (25 µM) and MG132 (20 µM) 3 hrs prior experiment and MoM cells were infected as mentioned in Fig 1. CFU was measured after 0 hr. B. TDMs were treated with Ca-074 (30 µM), SN50 (25 µM) and MG132 (20 µM) 3 hrs prior experiment and MoM cells were infected as mentioned in Fig 1. CFU was measured after 24 hrs. Error bars, means, standard deviations were done for three independent experiments, *** by students’ T Test. All statistical analysis was done in Graph Pad-Prism 8.
(PDF)

S21 Fig T4519 mediated LMP causes bacterial survival. A. MoM cells were incubated with dextran Alexa-fluor overnight and then treated with LLOMe (0.5 mM for 3hrs) to induce LMP. MoM cells were infected as mentioned in Fig 1 and treated with LLOMe (0.5 mM for 3hrs) at 12 hrs PI. B. MoM cells were infected as mentioned in Fig 1 and treated with LLOMe (0.5 mM for 3hrs) at 12 hrs PI. CFU was measured after 24 hrs. Error bars, means, standard deviations were done for three independent experiments, *** by students’ T Test. All statistical analysis was done in Graph Pad-Prism 8.
(PDF)

S22 Fig Sirt1 had no effect on Cathepsin B inhibition in Typhi infected cells. MoM cells were infected as mentioned in Fig 1. Western blot of Sirt1. β- actin was used as loading control for all the blots.
(PDF)

S1 Table List of primers used in this study. (PDF)

S2 Table Recipes for preparing pH calibration curve buffers. (PDF)

S1 Data (XLSX)
