# Supplementary figures and images for: Salmonella Typhi serine threonine kinase T4519 induces lysosomal membrane permeabilization by manipulating Toll-like receptor 2-Cystatin B-Cathepsin B-NF-κB-reactive oxygen species pathway and promotes survival within human macrophages
Source: PLoS Pathog. 2025 Apr 1;21(4):e1013041. doi: 10.1371/journal.ppat.1013041 (PMC11984733; doi:10.1371/journal.ppat.1013041)

S1 Fig

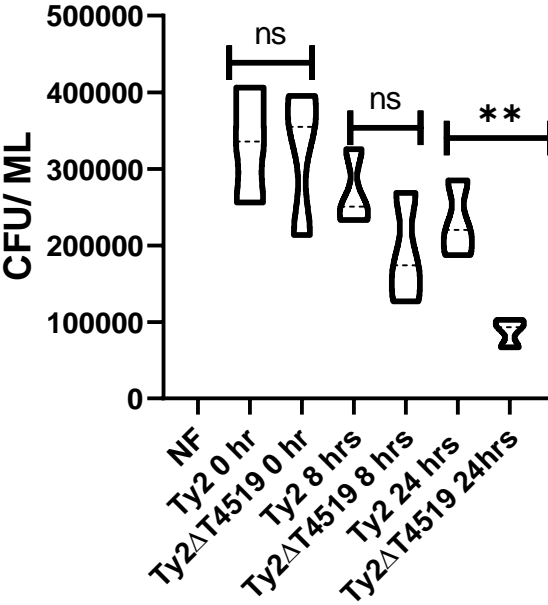

Supplement: S1 Fig — MoM cells were infected with wild type S. Typhi or Ty2ΔT4519 for 30 mins followed by gentamycin protection assay as described under Materials and Methods. CFU determined T4519 mediated survival. Intracellular CFU were counted after 24 hrs of infection following cell lysis and the lysates were platted on LA plates, which were incubated overnight at 37° C. Here P*< 0.05 compared between CFU of Ty2 24 hrs and ΔT4519Ty2 24 hrs. (PDF) [file ppat.1013041.s001.pdf]

S2 Fig

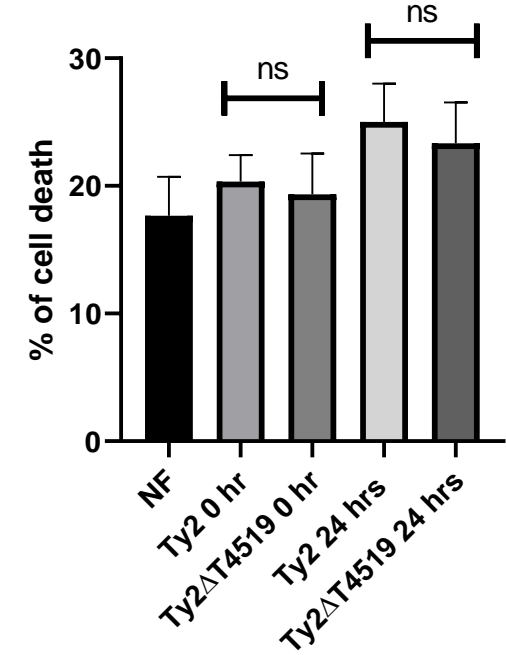

Supplement: S2 Fig — MoM cells were infected and LDH was measured from the culture supernatants of the cells at 24 hrs P.I. (PDF) [file ppat.1013041.s002.pdf]

S3 Fig

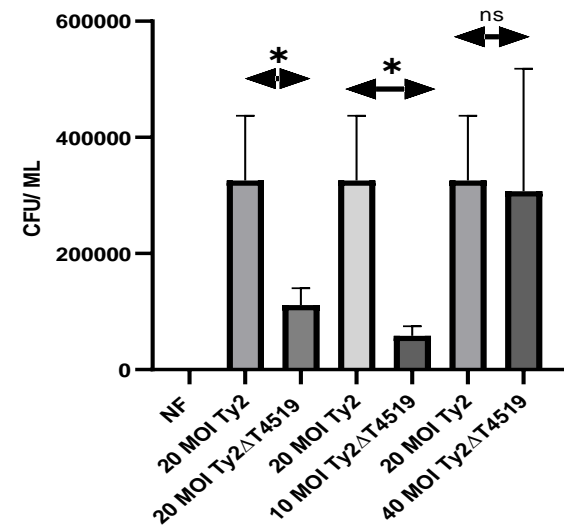

Supplement: S3 Fig — Here P*< 0.05 compared between CFU of Ty2 20 MoI and ΔT4519Ty2 20 MoI. Here P*<0.05 compared between CFU of Ty2 20 MoI and ΔT4519Ty2 10 MoI. Significance was calculated using two tailed unpaired T test. Statistical analysis was done by using GraphPad Prism 8. NS stands for not significant. (PDF) [file ppat.1013041.s003.pdf]

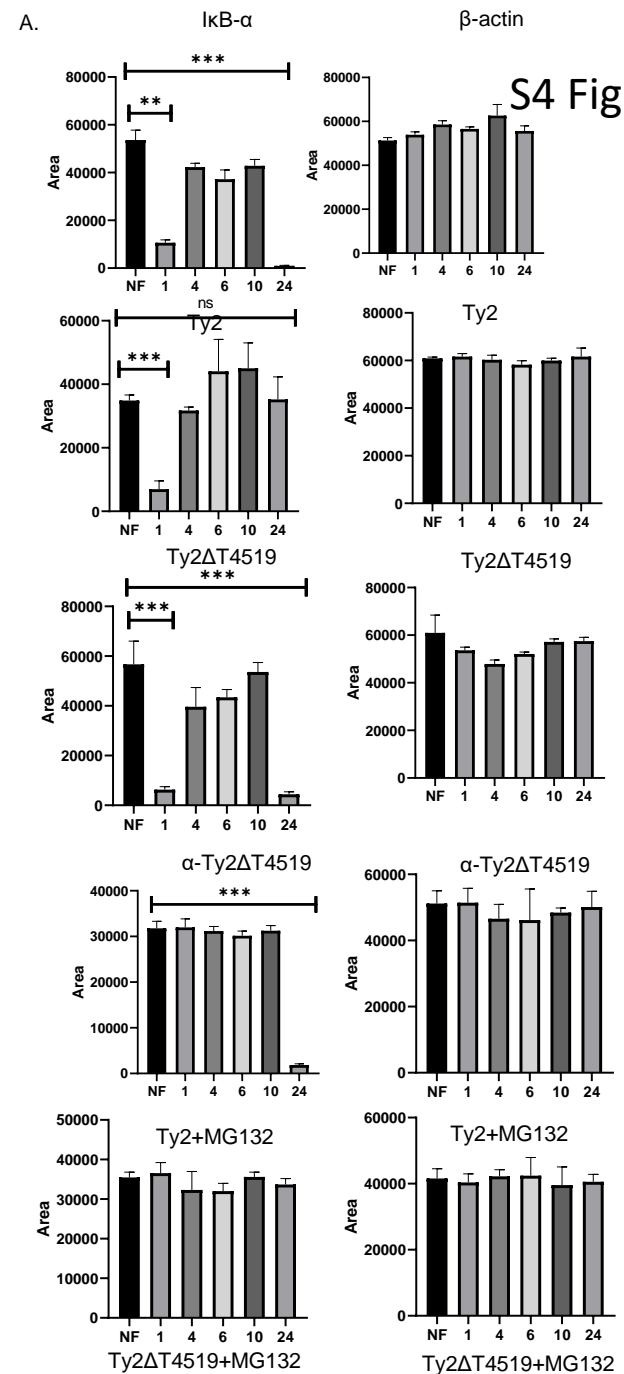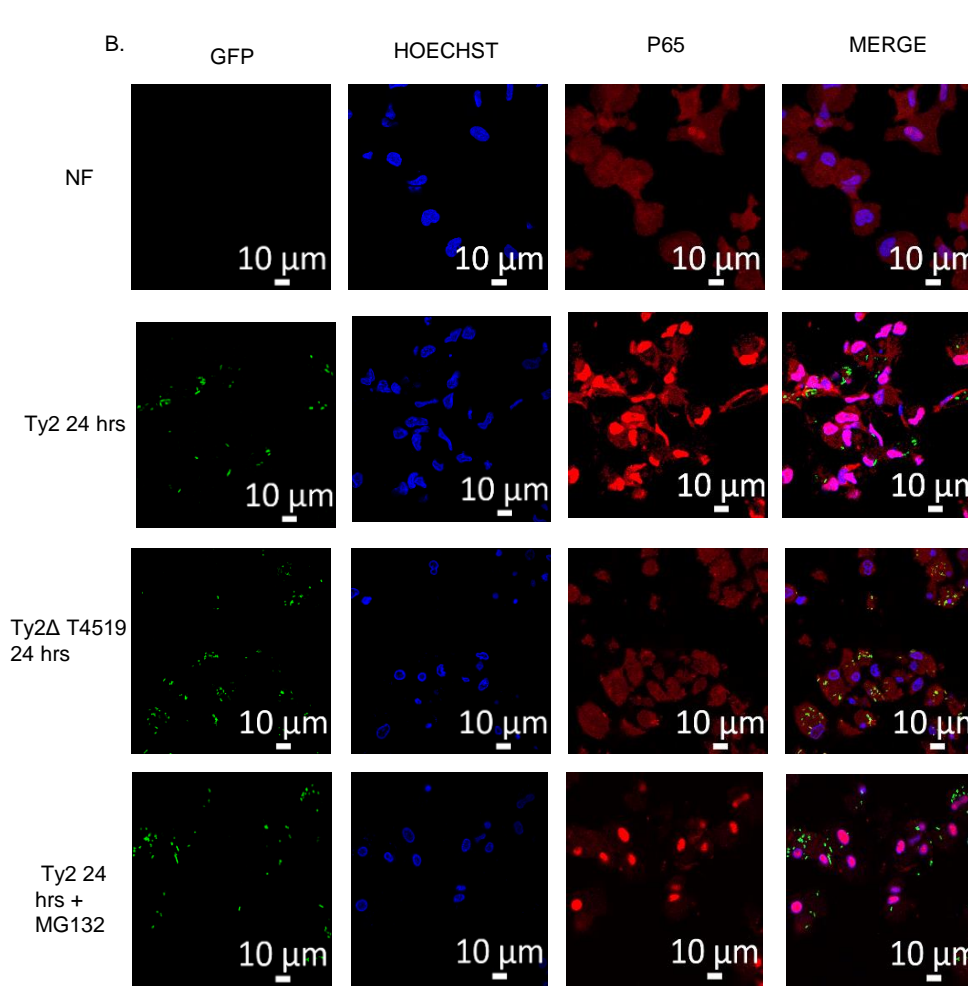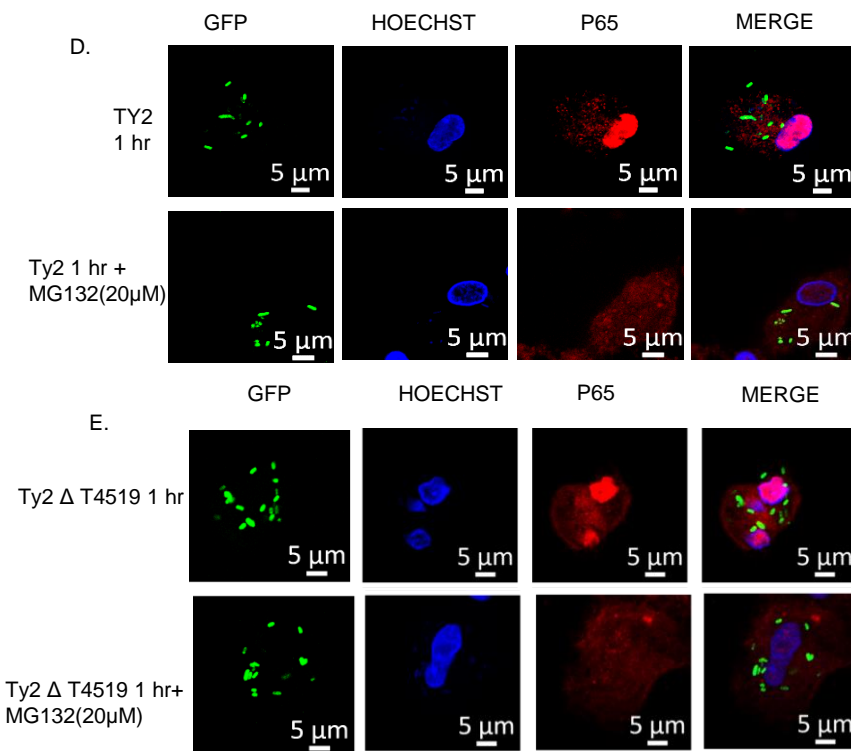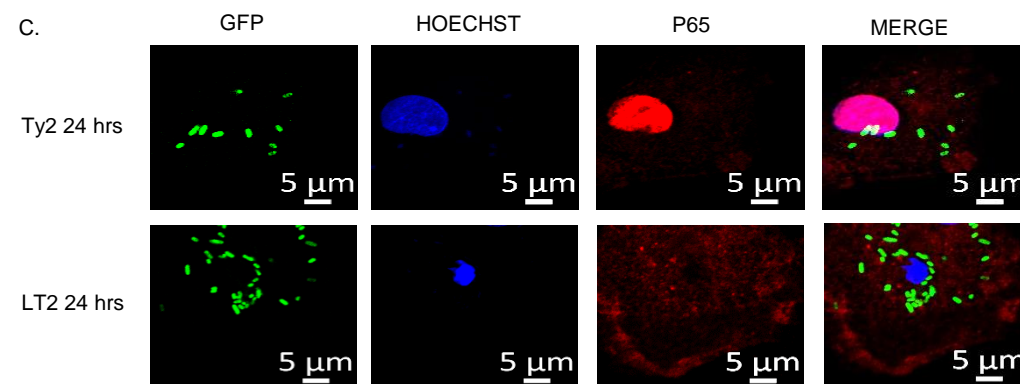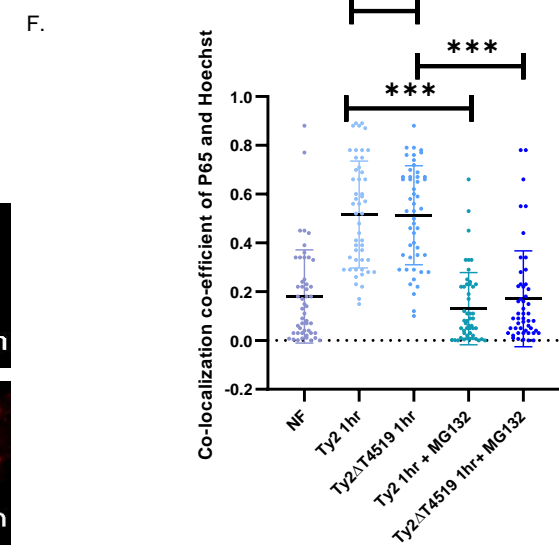

Supplement: S4 Fig — MoM cells were infected with wild type S. Typhi or Ty2ΔT4519 for 30 mins followed by gentamycin protection assay as described under Materials and Methods. A. Quantification of IκB-α and β-actin western blots. Quantification was done by using ImageJ software and graph is plotted in graph pad prism 8.B-D–p65 confocal microscopy staining after infection with GFP tagged bacteria. MoM cells were incubated with indicated inhibitors followed by infection. Alexa-fluor conjugated secondary antibody (Alexa-fluor 594 anti rabbit) was used for p65 staining and nucleus was stained by Hoechst (blue). Here NF stands for no Infection. The above experiments were repeated three times and the values from those three experiments were plotted. Error bars represent SD. Significance was calculated using two tailed unpaired T test. Statistical analysis was done by using GraphPad Prism 8. NS stands for not significant. A representative image from each experiment was given. E.Quantification of fluorescence colocalization of Hoechst (blue) and p65 (red), done by taking random fields, from which in a total of 50 cells, blue and red co-localization were measured by using LSM 710 Zeiss Zen Blue software of confocal microscope. P***< 0.001,compared between Ty2 1 hr and MG132 Ty2 1 hr infected cells and P***< 0.001, compared between Ty2ΔT4519 Ty2 1 hr and MG132 Ty2ΔT4519 1 hr infected cells. (PDF) [file ppat.1013041.s004.pdf]

S5 Fig

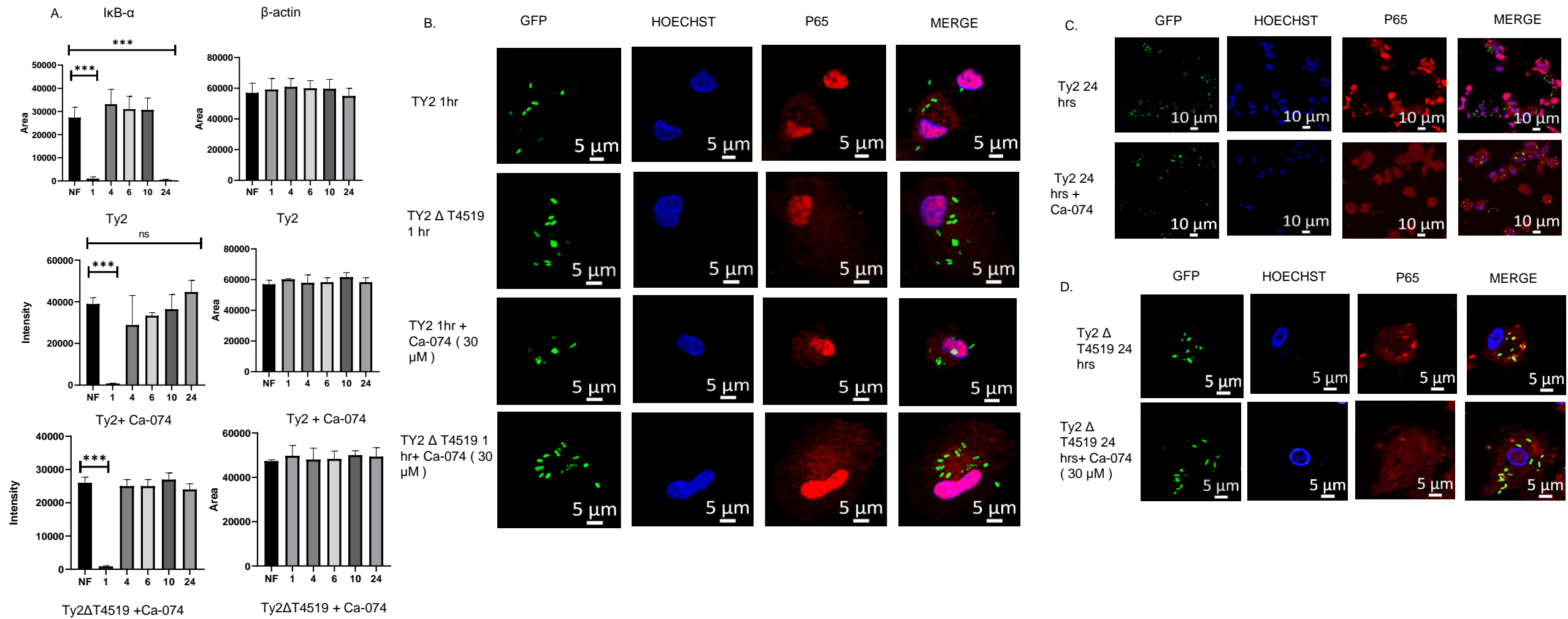

Supplement: S5 Fig — MoM cells were incubated with indicated inhibitors followed by infection. A. Quantification of IκB-α and β-actin western blots. Quantification was done by using ImageJ software and graph is plotted in graph pad prism 8. B-D. p65 confocal microscopy staining was done. Alexa-fluor conjugated secondary antibody (Alexa-fluor 594 anti rabbit) was used for p65 staining and nucleus was stained by Hoechst (blue). (PDF) [file ppat.1013041.s005.pdf]

S6 Fig

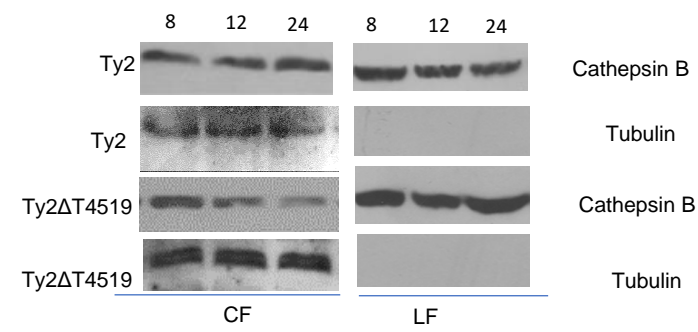

Supplement: S6 Fig — Infected MoM cells were lysed and cytoplasmic fraction (CF) and lysosomal fraction (LF) were isolated. Western blot was done by cathepsin B antibody at 8 hrs, 12 hrs and 24 hrs PI respectively. Tubulin was used as loading control. (PDF) [file ppat.1013041.s006.pdf]

S7 Fig

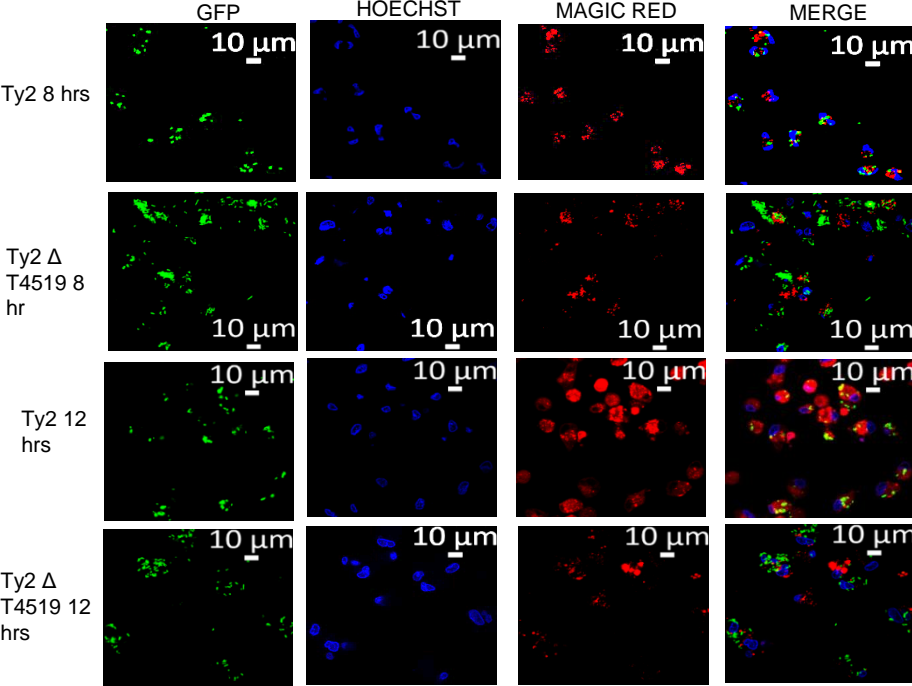

Supplement: S7 Fig — MoM cells were infected with GFP tagged bacteria and stained with magic red. Nucleus is stained with Hoechst. Representative images from each experiment was given. Images were taken in Zeiss LSM 710 confocal microscope. (PDF) [file ppat.1013041.s007.pdf]

S8 Fig

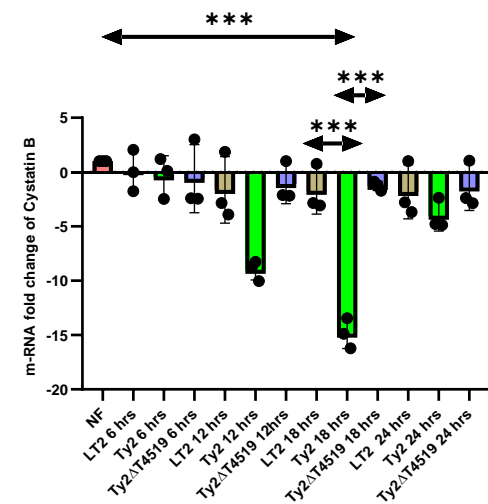

Supplement: S8 Fig — m-RNA fold changes of cystatin B measured by RT-Qpcr. β-actin was used as control gene. (PDF) [file ppat.1013041.s008.pdf]

S9 Fig

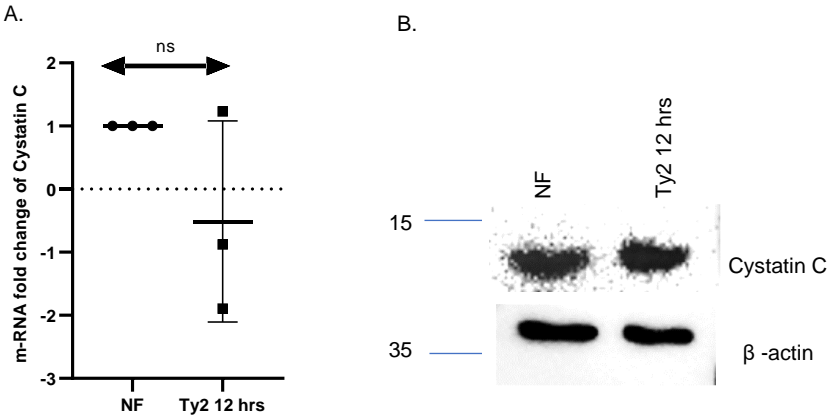

Supplement: S9 Fig — (A). m-RNA fold change of cystatin C was measured by RT-PCR. β-actin was used as control gene. (B). The western blot of cystatin C. The time points taken was 12 hrs PI. β- actin was used as loading control for all the blots. (PDF) [file ppat.1013041.s009.pdf]

S10 Fig

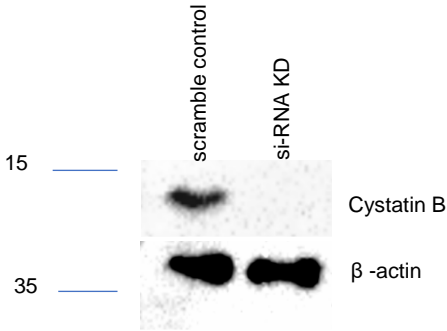

Supplement: S10 Fig — WB showing expression of cystatin B in Scramble Control and knockout cells. (PDF) [file ppat.1013041.s010.pdf]

S11 Fig

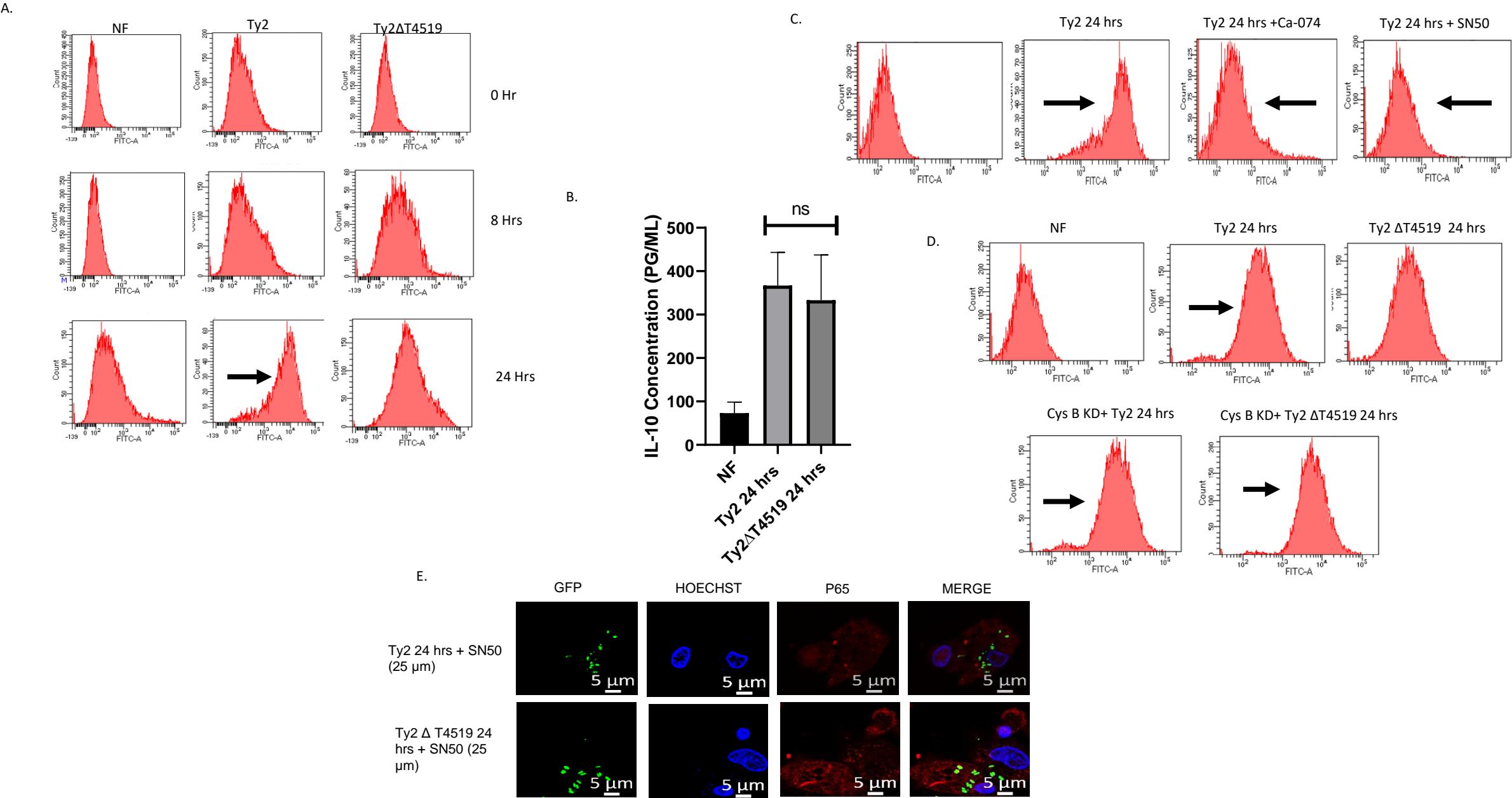

Supplement: S11 Fig — A,C,D. MoM cells were infected followed by staining with PBS containing 10 µM of CM-H₂DCFDA (Invitrogen) and FACS was done in FITC filter. NF stands for No Infection. B. ELISA to quantitate IL-10 concentration in MOM culture.C-D. Representative histogram images of FACS.The shift of MFI (Mean Fluorescent Intensity) of FITC peak was shown by an arrow. E. Nuclear translocation of p65 was hindered by SN-50 treatment proved by confocal microscopy. (PDF) [file ppat.1013041.s011.pdf]

S12 Fig

A.

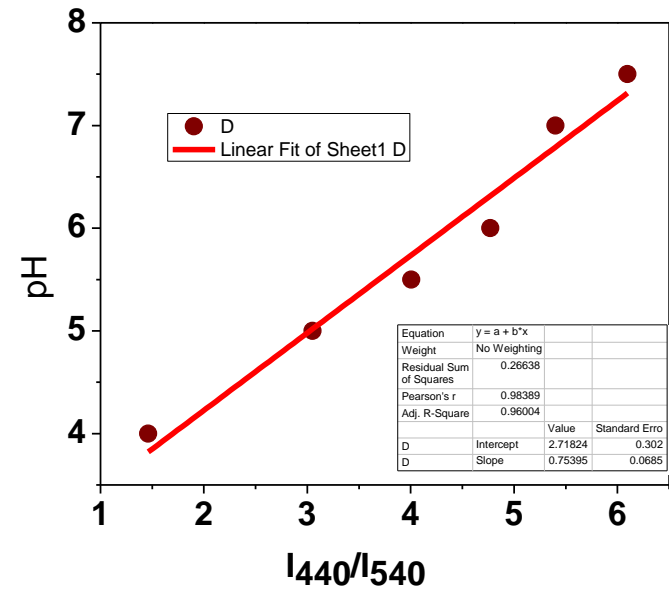

B.

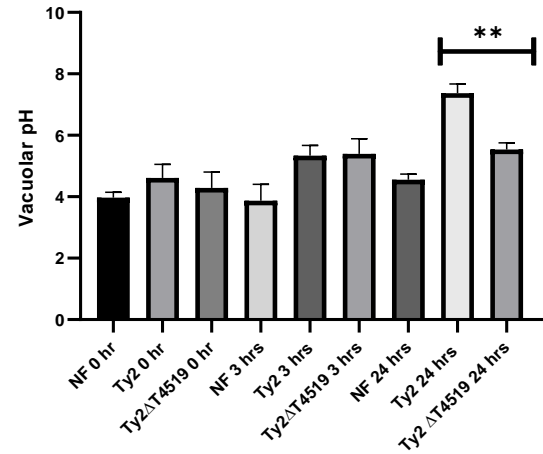

Supplement: S12 Fig — (A). MoM cells were stained with lysosensor yellow blue DND dye and exposed to different pH containing buffer from 3 to 8. Excitation and emission ratio of 440/540 was taken in a fluorimeter. The values were plotted to make the standard curve. (B). MoM cells were infected and then incubated in lysosensor yellow blue DND dye and excitation and emission ratio of 440/540 was taken in a fluorimeter. The value was plotted in the standard curve to obtain the correct ph. Graphical representation of different pH was given. (PDF) [file ppat.1013041.s012.pdf]

S13 Fig

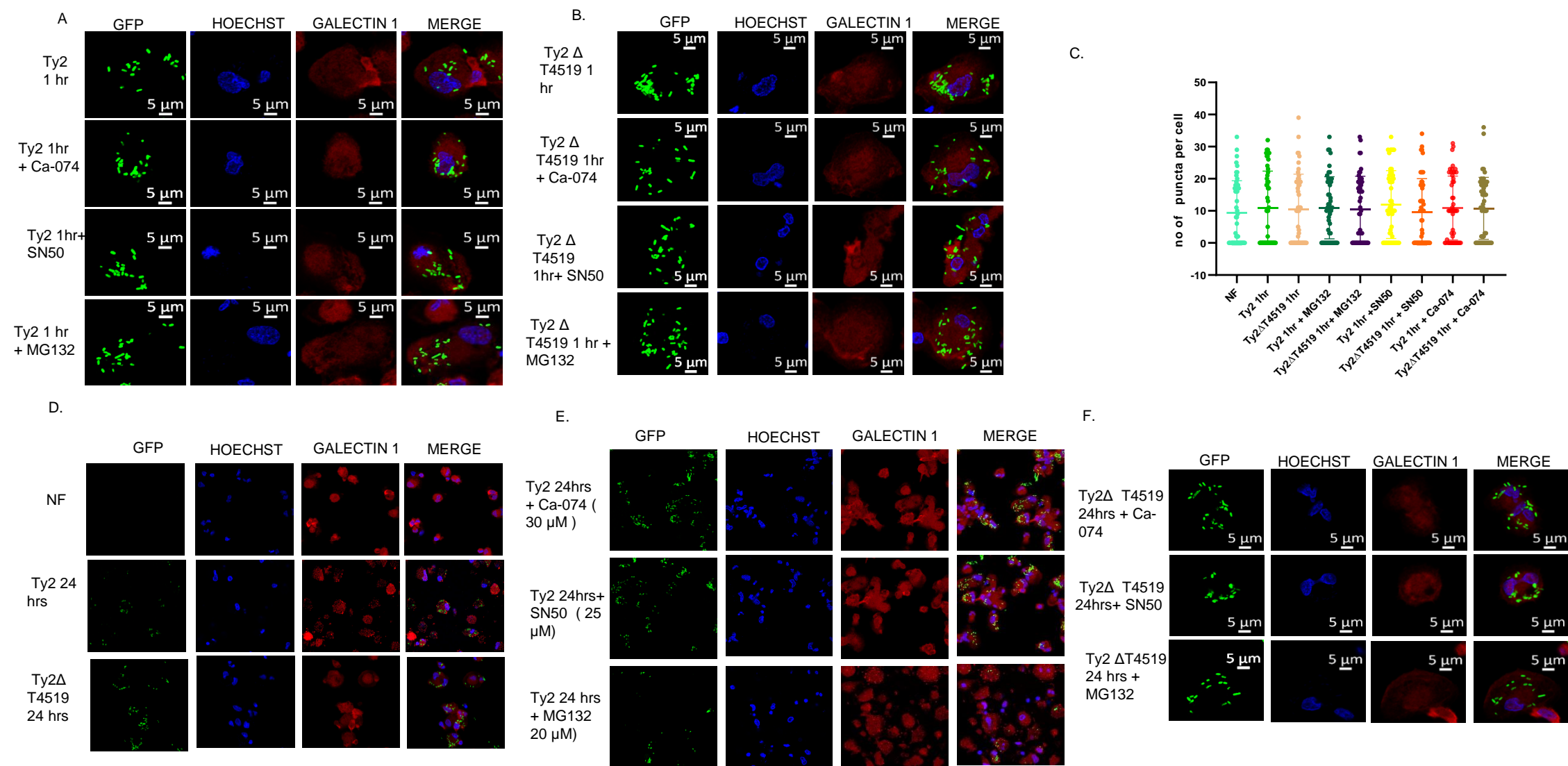

Supplement: S13 Fig — (A-F). MoM cells were infected and stained with Galectin 1 antibody followed by Alexa-fluor conjugated secondary antibody and nucleus was stained by Hoechst (blue). (C). Quantification of Galectin-1 puncta was done by Fiji ImageJ software “particle analysis” tool. Non-punctas were eliminated manually. Cells were randomly selected from each field. N=50 cells., under LSM 710 ZEISS confocal microscope. A representative image from all experiments was given. (PDF) [file ppat.1013041.s013.pdf]

S14 Fig

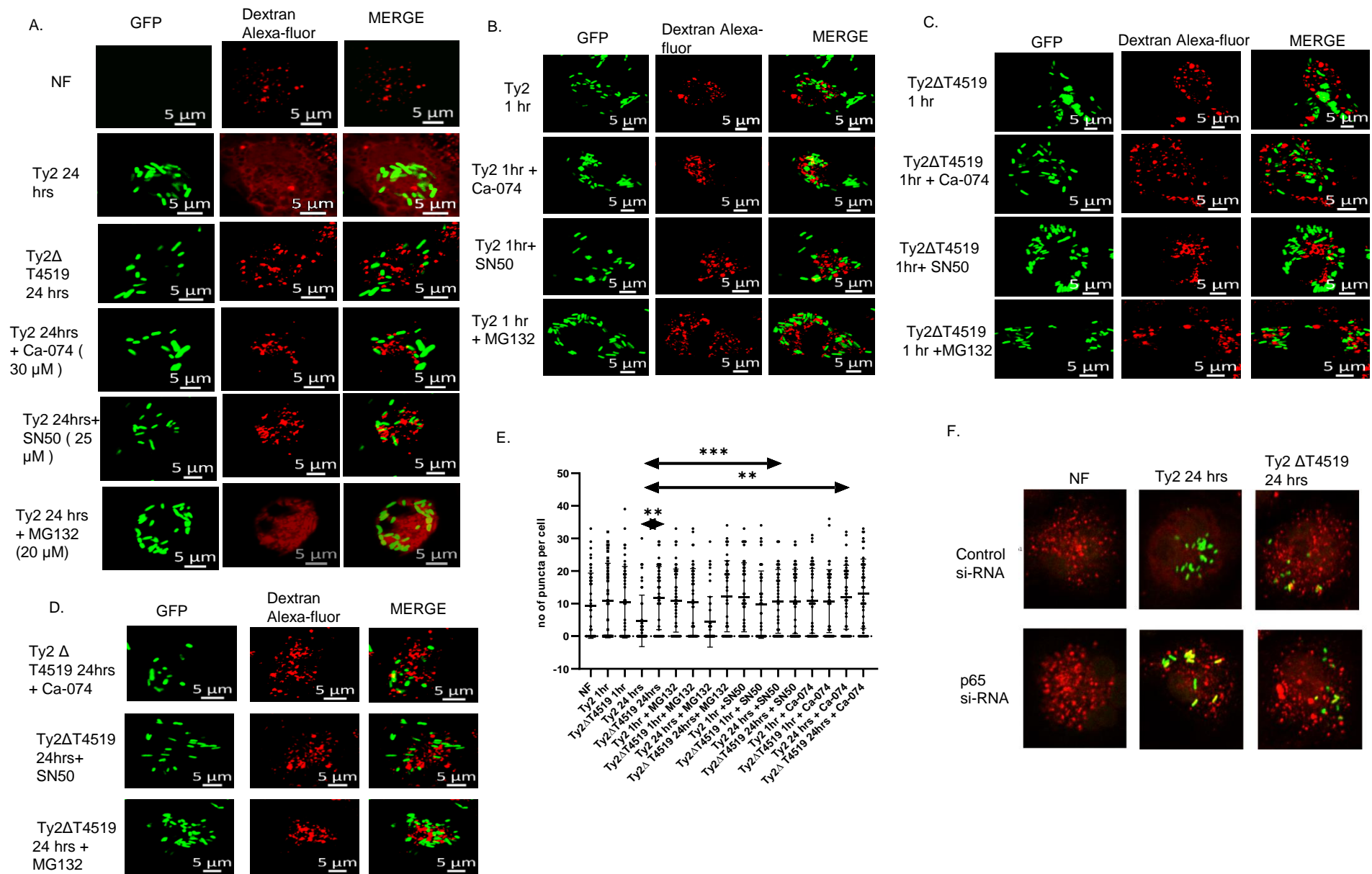

Supplement: S14 Fig — (A-E). Cells were incubated with Dextran Alexa-fluor containing media overnight and then infected with GFP tagged bacteria. (E). Quantification of dextran puncta. N=50 Cells (F). Lysotracker red staining after infecting p65 si-RNA KD TDM cells. TDM cells were treated with p65 si-RNA to knock down p65 followed by infection. (PDF) [file ppat.1013041.s014.pdf]

S15 Fig

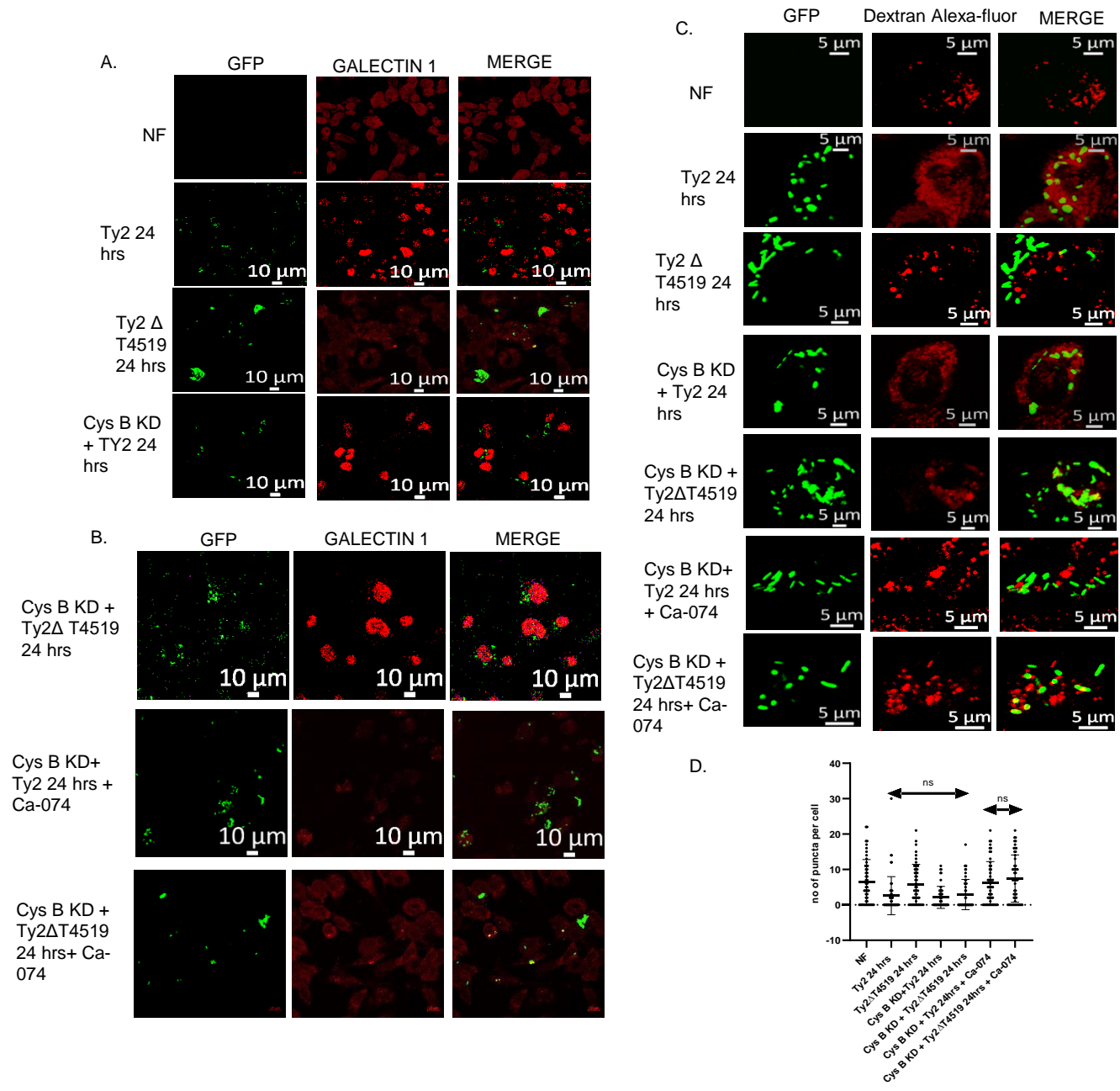

Supplement: S15 Fig — (A-D). Cystatin B KD was done by si-RNA. (A-B). Cystatin B KD TDM cells were infected and stained with Galectin 1 antibody followed by Alexa-fluor conjugated secondary antibody (C). THP-1 derived macrophages were incubated with Dextran Alexa-fluor containing media overnight and then infected with bacteria. (D). Quantification of dextran puncta was done by Fiji ImageJ software “particle analysis” tool. Non-punctas were eliminated manually. Cells were randomly selected from each field. N= 50 cells. Representative images from all these experiments were given. Confocal images were taken in LSM 710 confocal microscope and statistical analysis were done in Graph Pad-Prism 8 by unpaired Students’ T-test. (PDF) [file ppat.1013041.s015.pdf]

S16 Fig

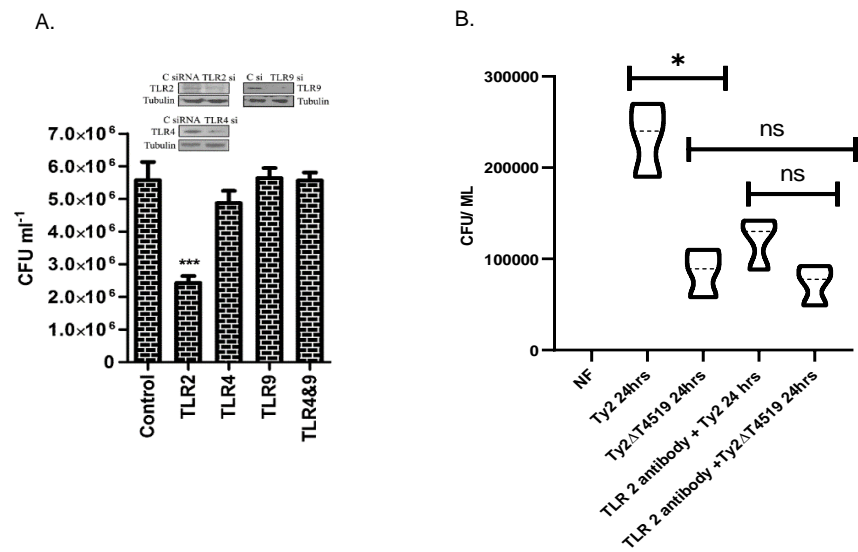

Supplement: S16 Fig — (A). CFU of wild type bacteria after infecting TDM cells at 24 hrs. TDM cells were treated with different si-RNA of that particular TLR to knock down. Knock down was tested by western blot. Tubulin was used as loading control. (B). CFU of wild type and mutant bacteria at 24 hrs PI after lysing MoM cells. Error bars, means, standard deviations were done for three independent experiments, *** by students’ T Test. All statistical analysis was done in Graph Pad-Prism 8. (PDF) [file ppat.1013041.s016.pdf]

S17 Fig

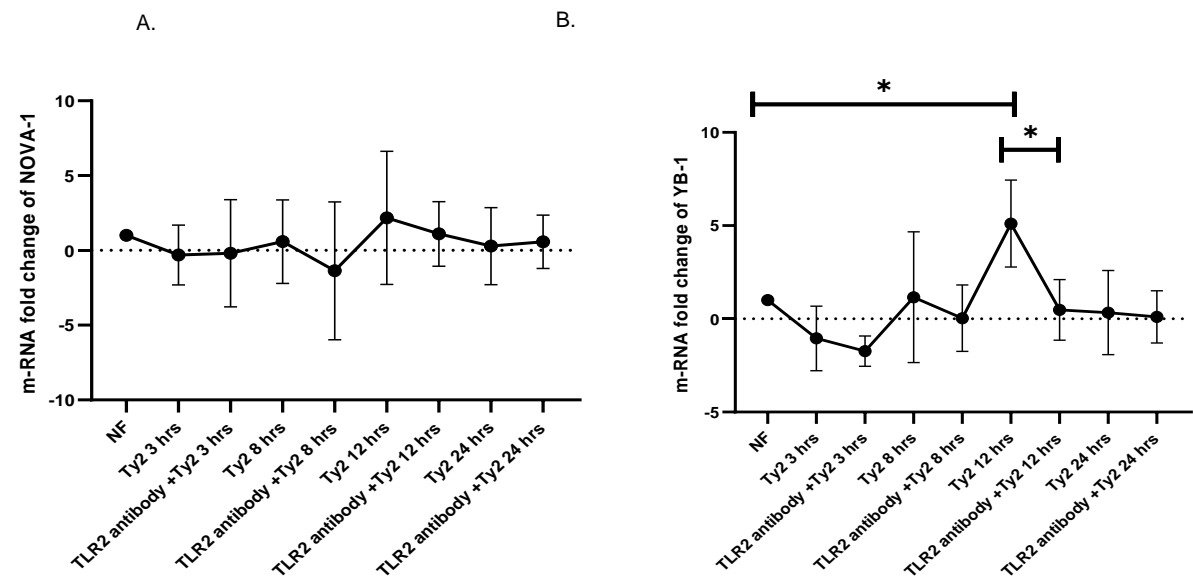

Supplement: S17 Fig — (A-B). m-RNA expression was measured by RT-PCR after infecting the MoM cells with Ty2. (A). m-RNA fold change of NOVA-1, (B). m-RNA fold change of YB-1. Error bars, means, standard deviations were done for three independent experiments, *** by students’ T Test. All statistical analysis was done in Graph Pad-Prism 8. (PDF) [file ppat.1013041.s017.pdf]

S18 Fig

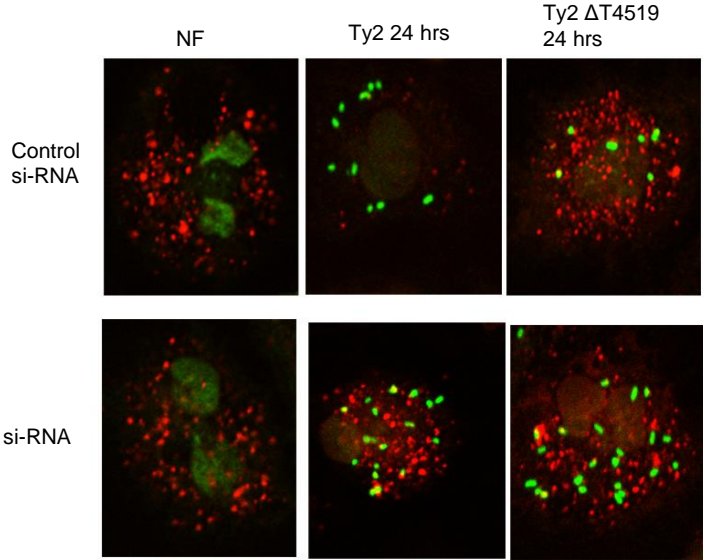

Supplement: S18 Fig — Lysotracker red staining after 24 hrs of infection in TDM cells. TDM cells were treated with TLR2 si-RNA to knock down TLR-2 before infection with GFP tagged bacteria. A representative image of all experiments were given. Image captured in Zeiss LSM 710 confocal microscope. (PDF) [file ppat.1013041.s018.pdf]

S19 Fig

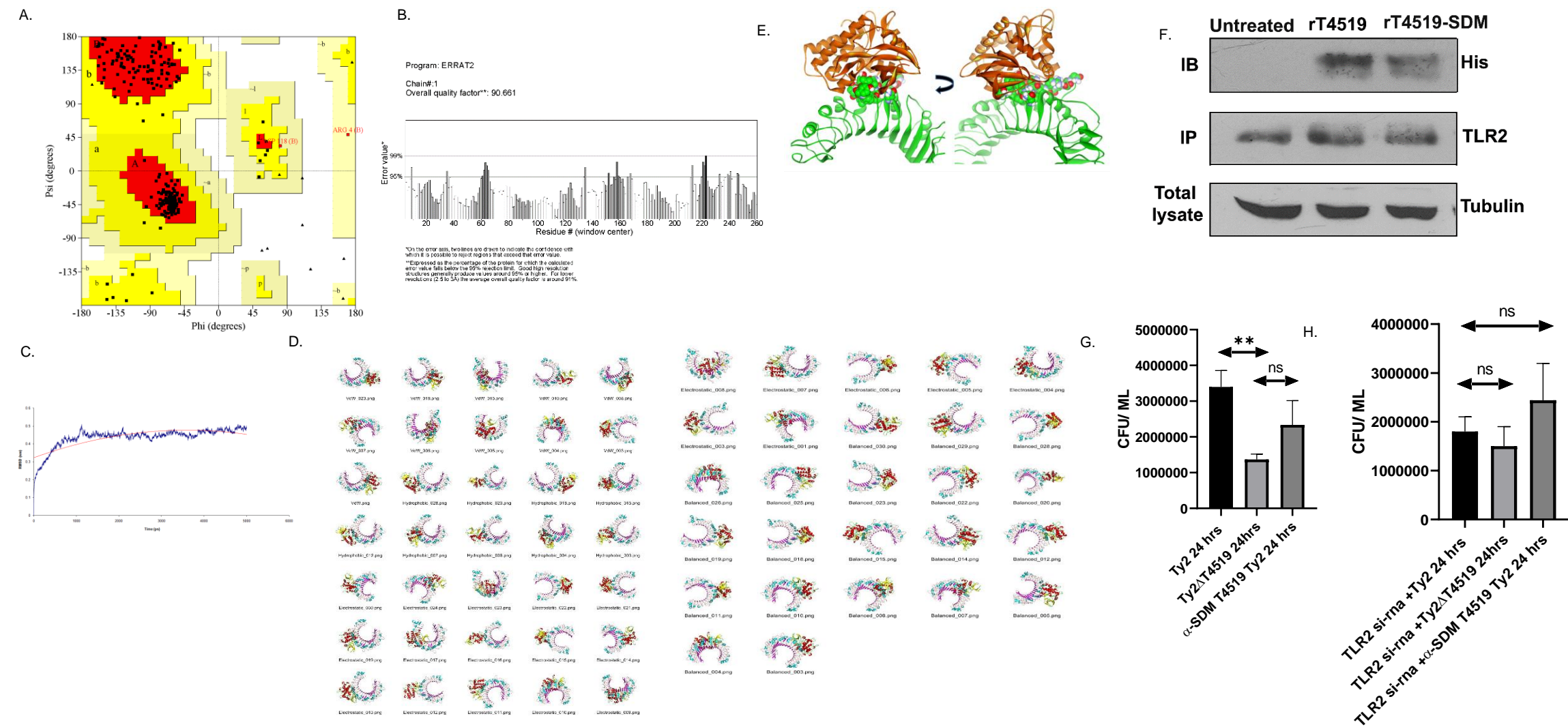

Supplement: S19 Fig — (A-E). Homology modeling of T4519. (A). Ramachandran plot of the 3D model structure of T4519. Ramachandran plot for the t4519 model structure shows 90.6% of the residues are in the allowed regions whereas 8.6% and 0.9% residues are observed in the additional and generously allowed regions, respectively (B). ERRAT plot shows the statistics of non-bonded interactions between different atoms. The overall quality of the modeled protein structure is indicated by the score of 90.661 (Low resolution structures produce values around 91). (C). 5 nanoseconds molecular dynamics simulation plot of T4519 showing relative deviation of the protein backbone atoms during simulation. Polynomial curve (Red) shows that the protein is quite stable after 2 ns as it fluctuates within the range of 0.4-0.5 nm. D. 62 interactions between TLR2 and T4519 produced by ClusPro 2.0 docking server. Figure represents favored interactions, which include 11 Vanderwalls, 9 Hydrophobic, 22 Electrostatic and 20 balanced interactions. E. The cartoon representation of t4519 and TLR2 complex. The image showing two loops from TLR2 (in CPK) positioned between N and C terminal hydrophobic region of t4519. F. Stable expression TLR2/6 HEK293 cells were transiently transfected with TLR accessory proteins. Cells were stimulated with rT4519, rT4519-SDM (G12A/K13V/G14A) for 24 hrs and immunoprecipitation were done. (G-H). Cells were infected with the indicated bacterial strains. CFU counts of intracellular bacteria recovered from the lysed cells after 24 hrs were plotted. (PDF) [file ppat.1013041.s019.pdf]

S20 Fig

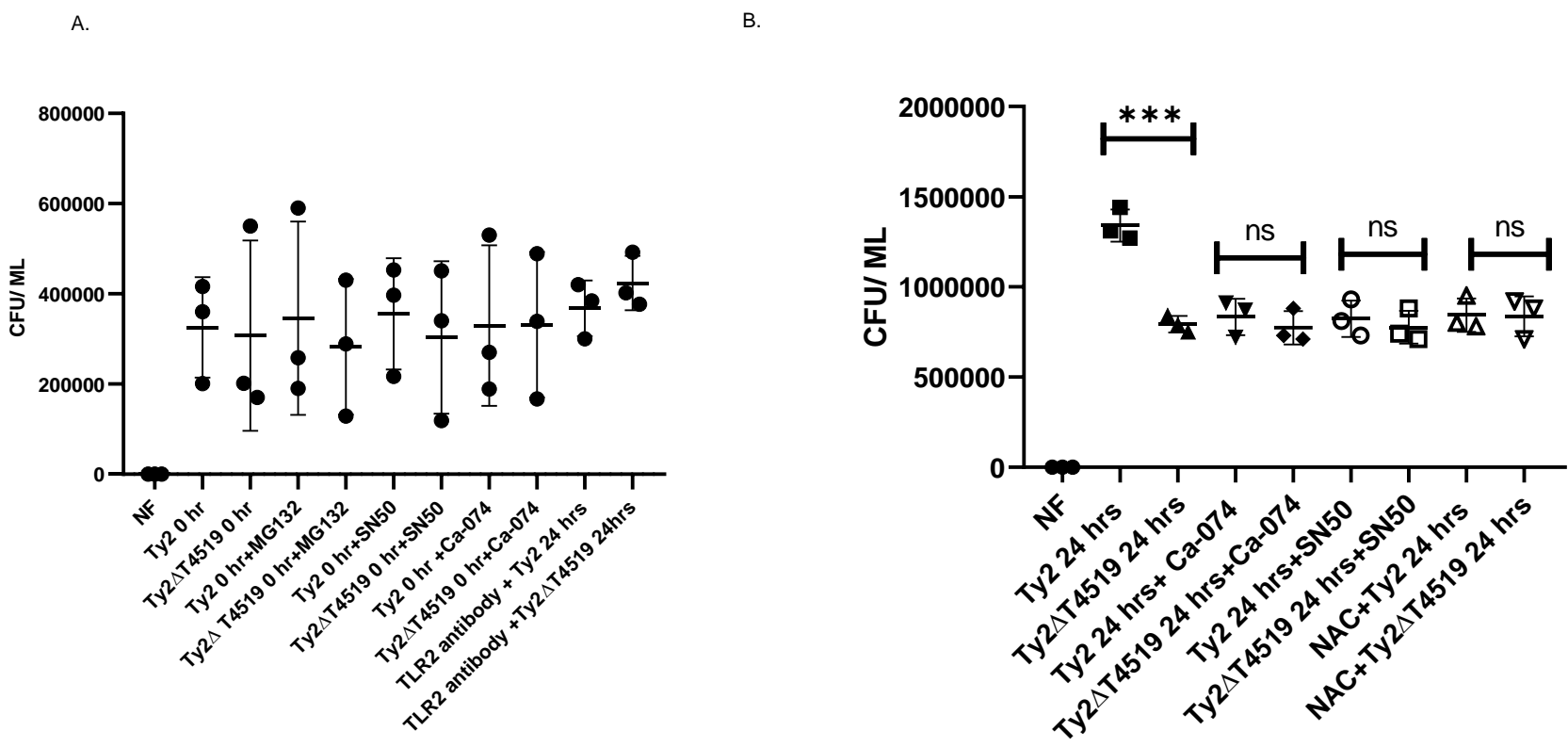

Supplement: S20 Fig — Bacterial CFU at 0 hr time point after infection shows no significant difference. MoM cells were treated with Ca-074 (30 µM), SN50 (25 µM) and MG132 (20 µM) 3 hrs prior experiment and MoM cells were infected as mentioned in Fig 1. CFU was measured after 0 hr. B. TDMs were treated with Ca-074 (30 µM), SN50 (25 µM) and MG132 (20 µM) 3 hrs prior experiment and MoM cells were infected as mentioned in Fig 1. CFU was measured after 24 hrs. Error bars, means, standard deviations were done for three independent experiments, *** by students’ T Test. All statistical analysis was done in Graph Pad-Prism 8. (PDF) [file ppat.1013041.s020.pdf]

S21 Fig

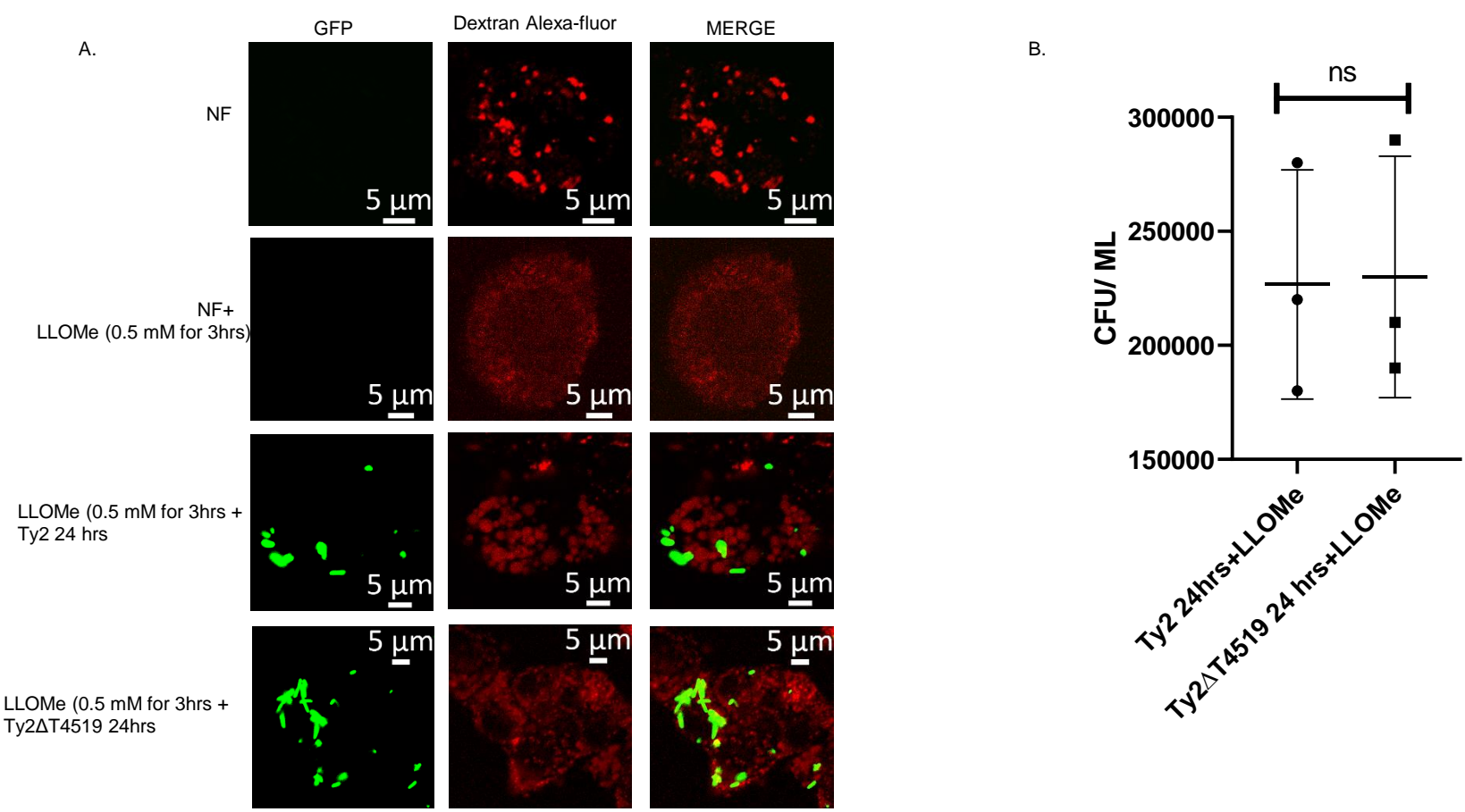

Supplement: S21 Fig — A. MoM cells were incubated with dextran Alexa-fluor overnight and then treated with LLOMe (0.5 mM for 3hrs) to induce LMP. MoM cells were infected as mentioned in Fig 1 and treated with LLOMe (0.5 mM for 3hrs) at 12 hrs PI. B. MoM cells were infected as mentioned in Fig 1 and treated with LLOMe (0.5 mM for 3hrs) at 12 hrs PI. CFU was measured after 24 hrs. Error bars, means, standard deviations were done for three independent experiments, *** by students’ T Test. All statistical analysis was done in Graph Pad-Prism 8. (PDF) [file ppat.1013041.s021.pdf]

S22 Fig

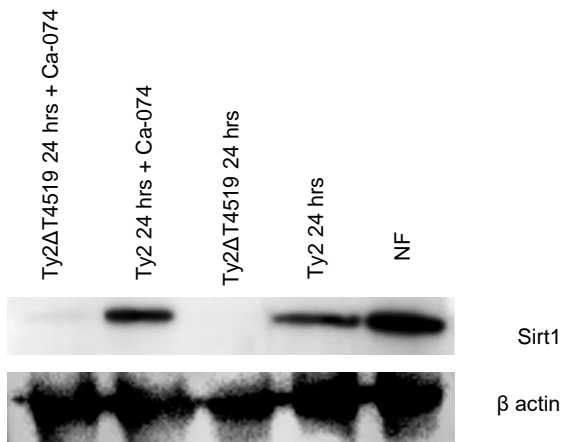

Supplement: S22 Fig — MoM cells were infected as mentioned in Fig 1. Western blot of Sirt1. β- actin was used as loading control for all the blots. (PDF) [file ppat.1013041.s022.pdf]
