# Supplementary material for: Salmonella Typhi serine threonine kinase T4519 induces lysosomal membrane permeabilization by manipulating Toll-like receptor 2-Cystatin B-Cathepsin B-NF-κB-reactive oxygen species pathway and promotes survival within human macrophages
Source: PLoS Pathog. 2025 Apr 1;21(4):e1013041. doi: 10.1371/journal.ppat.1013041 (PMC11984733; doi:10.1371/journal.ppat.1013041)
Supplement: S1 Table — (PDF) [file ppat.1013041.s023.pdf]

Primer Name	5'<-----Sequence----->3'
Cystatin B forward primer	5’CTGTGTTTAAGGCCGTGTCA3’
Cystatin B reverse primer	5’AGGTCAGCTCATCATGCTTG3’
Cystatin C forward primer	5’GCGGCGTGCACTGGACTTTG3’
Cystatin C reverse primer	5’GCCGCCTGCTGCCTTCTCTG3’
β-Actin forward primer	5’ACAATGTGGCCGAGGACTTT3’
β-Actin reverse primer	5’GCACGAAGGCTCATCATTCA3’
NOVA1 forward primer	5’TTGCCATCTTCCCCAACTAC3’
NOVA1 reverse primer	5’ TTACAGCCTTCACAGTAGCAC3’
YB-1 forward primer	5′-AAGTGATGGAGGGTGCTGAC-3′
YB-1 reverse primer	5′-TTCTTCATTGCCGTCCTCTC-3′
