# Supplementary material for: Salmonella Typhi serine threonine kinase T4519 induces lysosomal membrane permeabilization by manipulating Toll-like receptor 2-Cystatin B-Cathepsin B-NF-κB-reactive oxygen species pathway and promotes survival within human macrophages
Source: PLoS Pathog. 2025 Apr 1;21(4):e1013041. doi: 10.1371/journal.ppat.1013041 (PMC11984733; doi:10.1371/journal.ppat.1013041)
Supplement: S2 Table — (PDF) [file ppat.1013041.s024.pdf]

	1M KCl	5M NaCl	72mM Monensin	0.5M HEPES	0.5M MES	1N NaOH*	1N HCl*	H_2O
Buffer of pH 7.5	6.25 mL	0.25 mL	6.9 µL	2.5 mL	-	*	*	To 50 mL
Buffer of pH 7.0	6.25 mL	0.25 mL	6.9 µL	2.5 mL	-	*	*	to 50 mL
Buffer of pH 6.5	6.25 mL	0.25 mL	6.9 µL	-	2.5 mL	*	*	To 50 mL
Buffer of pH 6	6.25 mL	0.25 mL	6.9 µL	-	2.5 mL	*	*	To 50 mL
Buffer of pH 5.5	6.25 mL	0.25 mL	6.9 µL	-	2.5 mL	*	*	to 50 mL
Buffer of pH 5	6.25 mL	0.25 mL	6.9 µL	-	2.5 mL	*	*	to 50 mL
Buffer of pH 4.5	6.25 mL	0.25 mL	6.9 µL	-	2.5 mL	*	*	to 50 mL
Buffer of pH 4	6.25 mL	0.25 mL	6.9 µL	-	2.5 mL	*	*	to 50 mL
Buffer of pH 3.5	6.25 mL	0.25 mL	6.9 µL	-	2.5 mL	*	*	to 50 mL
